# TRESK Background K^+^ Channel Is Inhibited by PAR-1/MARK Microtubule Affinity-Regulating Kinases in Xenopus Oocytes

**DOI:** 10.1371/journal.pone.0028119

**Published:** 2011-12-01

**Authors:** Gabriella Braun, Balázs Nemcsics, Péter Enyedi, Gábor Czirják

**Affiliations:** Department of Physiology, Semmelweis University, Budapest, Hungary; Sackler Medical School, Tel Aviv University, Israel

## Abstract

TRESK (TWIK-related spinal cord K^+^ channel, KCNK18) is a major background K^+^ channel of sensory neurons. Dominant-negative mutation of TRESK is linked to familial migraine. This important two-pore domain K^+^ channel is uniquely activated by calcineurin. The calcium/calmodulin-dependent protein phosphatase directly binds to the channel and activates TRESK current several-fold in *Xenopus* oocytes and HEK293 cells. We have recently shown that the kinase, which is responsible for the basal inhibition of the K^+^ current, is sensitive to the adaptor protein 14-3-3. Therefore we have examined the effect of the 14-3-3-inhibited PAR-1/MARK, microtubule-associated-protein/microtubule affinity-regulating kinase on TRESK in the *Xenopus* oocyte expression system. MARK1, MARK2 and MARK3 accelerated the return of TRESK current to the resting state after the calcium-dependent activation. Several other serine-threonine kinase types, generally involved in the modulation of other ion channels, failed to influence TRESK current recovery. MARK2 phosphorylated the primary determinant of regulation, the cluster of three adjacent serine residues (S274, 276 and 279) in the intracellular loop of mouse TRESK. In contrast, serine 264, the 14-3-3-binding site of TRESK, was not phosphorylated by the kinase. Thus MARK2 selectively inhibits TRESK activity via the S274/276/279 cluster, but does not affect the direct recruitment of 14-3-3 to the channel. TRESK is the first example of an ion channel phosphorylated by the dynamically membrane-localized MARK kinases, also known as general determinants of cellular polarity. These results raise the possibility that microtubule dynamics is coupled to the regulation of excitability in the neurons, which express TRESK background potassium channel.

## Introduction

TRESK is abundantly expressed in dorsal root ganglion (DRG) neurons and has been suggested to play an important role in pain disorders [1]–[5]. TRESK is the target of sanshool, the paresthetic and counter-irritant ingredient of the traditional Chinese medicine, Sichuan pepper [6], [7]. The channel has recently attracted particular attention, because its dominant-negative mutation was reported to be linked to familial migraine with aura [8]. These findings indicate the importance of TRESK in pain control and points to the need for better understanding of the regulatory properties of the channel.

TRESK regulation is distinguished within the K2P channel family by the unique sensitivity to the cytoplasmic calcium signal. The calcium/calmodulin-dependent protein phosphatase calcineurin activates TRESK 5–15-fold in *Xenopus* oocytes [9]. Stimulation of G_q_ protein-coupled receptors activated TRESK by 40–80% in COS-7 cells under whole-cell patch clamp conditions [10], [11]. Whole-cell TRESK current in native cells has not been reliably measured, although several studies examined TRESK in isolated DRG neurons [5], [8], [10]–[13]. In the absence of specific inhibitors, separation of native whole-cell TRESK current from the other endogenous background K^+^ currents remains a challenge to be solved in the future. When cell-attached patches containing TRESK channels were painstakingly selected from DRG neurons, single channel activity increased by 30–80% in response to receptor stimulation [11]. The mechanism of TRESK activation in mammalian cells, and the cause of the apparently smaller stimulation of the current in the mammalian cell lines than in the *Xenopus* system have not yet been investigated.

We have recently realized that two inhibitory kinase pathways converge on TRESK [14]. The two pathways have different target residues in the intracellular loop of the channel. Protein kinase A phosphorylates the second serine in the conserved RSNSCPE sequence (S264 in mouse and S252 in human TRESK), thereby recruits the adaptor protein 14-3-3 to this motif [15], and exerts auxiliary channel inhibition [14]. However, the major inhibitory pathway targets the S274/276/279 cluster; RLSCSILSNLD in the mouse, corresponding to RLSYSIISNLD (S262/264/267) in human TRESK. Intriguingly, this pathway was shown to be inhibited by 14-3-3 even if the direct binding of the adapter protein to TRESK was abrogated [14]. The major aim of our present study was to identify the kinase, which phosphorylates the S274/276/279 cluster and accordingly inhibits TRESK, when expressed in the *Xenopus* oocyte system.

## Materials and Methods

### Plasmids and reagents

The cloning of human and mouse TRESK cDNAs [9] and S264E mutant mouse TRESK [14] were previously described. Mouse TRESK was subcloned to pIRES-CD8 vector [16] for transfection of HEK293 cells. Human embryonic kidney (HEK293) cell line (ATCC-CRL-1573) was purchased from LGC Standards GmbH (Wesel, Germany). The AMPK-related kinase and tau cDNAs were amplified with RT-PCR. Total RNAs were purified with TRIzol reagent (Invitrogen, Carlsbad, CA). Reverse transcription was performed with MMLV-RT (Revertaid, Fermentas, Vilnius, Lithuania) from mouse brain (BRSK1, MARK1, MARK2, MARK3, MARK4, NUAK1, tau), embryo body (SIK1(1–343)), testis (AMPKα1) or placenta (MELK) total RNAs. MARK1 and MELK PCR products were amplified with Ultra Pfu (Stratagen, La Jolla, CA), while those of the other kinases with Pfu polymerase (Fermentas). We have cloned isoform 2 of MARK2 (722 amino acids, Genebank NP_001073857), and used this protein throughout the study. For primer sequences, cloning sites and PCR protocols see Figure S1. All kinase cDNAs were cloned to pXEN vector (Genebank EU267939), and verified by automatic sequencing.

Different mutant versions of the kinases were produced with QuikChange site directed mutagenesis (Stratagen). For primer sequences see Figure S1. MARK2 T208E or T208E/T539A mutants were also subcloned into pGEX2TK4T1 and pET32-ΔKpn [15] vectors for the production of GST- or Trx-His_6_-tagged versions of the kinase in *E. coli*. (In pGEX2TK4T1 the EcoRI-PstI fragment of pGEX-4T-1 was cloned into pGEX-2TK, Amersham Biosciences, Little Chalfont, UK.) Tau coding sequence was cloned into pGEX-4T-1. Cloning and purification of GST-TRESKloop, GST-TRESKloop-TAPtag and different versions of TRESKloop-His_8_ protein were previously described [15], [17]. These proteins are hydrophobic; they are insoluble under non-denaturing conditions. Therefore, after prurification from bacterial lysates they were kept immobilized on the affinity matrix (on glutathione or Ni-NTA resins) and they were added in this form to the kinase reaction.

Ionomycin (calcium salt, Sigma) and FK506 were dissolved in DMSO as 5 mM stock solutions, and diluted to 0.5 or 1 µM before the measurement. Chemicals of analytical grade were purchased from Sigma, Fluka or Merck. Enzymes and kits of molecular biology applications were purchased from Ambion (Austin, TX), Fermentas, New England Biolabs (Beverly, MA), and Stratagene.

### Production and purification of recombinant MARK2 proteins

Glutathione S-transferase (GST) fusion constructs of constitutively active MARK2 mutants and tau were expressed in BL21 strain of *E. coli*. Solution A contained 50 mM Tris-HCl (pH 7.5), 200 mM NaCl, 1 mM β-mercaptoethanol, 1 mM PMSF and 2 mM benzamidine. Bacteria were sonicated in solution A supplemented with 5 mM CHAPS. GST fusion proteins were affinity-purified with glutathione-agarose (Sigma). GST-tau protein immobilized on glutathione-agarose was stored as a 50% suspension in solution A at 4°C. GST-MARK2 constructs were eluted from the resin with solution A containing 20 mM reduced glutathione.

The bacteria expressing the thioredoxin-His-tag (Trx-His_6_) fusion proteins were lysed in solution A supplemented with 15 mM imidazole and 5 mM CHAPS. The proteins were affinity-purified with Ni-NTA agarose (Qiagen, Chatsworth, CA). The resin was washed 2 times with solution A, and 3 times with solution A containing 60 mM imidazole. The protein was eluted with solution A supplemented with 300 mM imidazole. Both GST-MARK2 and Trx-His_6_-MARK2 enzymes were dialyzed against solution A containing 50% glycerol, and stored at −20°C.

### Animals, tissue preparation, *Xenopus* oocyte microinjection

Mouse tissues derived from NMRI mouse strain (Toxicop, Hungary). *Xenopus* oocytes were prepared, the cRNA was synthesized and microinjected as previously described [9]. Oocytes were microinjected one day after defolliculation. Fifty nanoliters of the appropriate RNA solution was delivered with Nanoliter Injector (World Precision Instruments, Saratosa, Florida, USA). All treatments of the animals were conducted in accordance with state laws and institutional regulations. The experiments were approved by the Animal Care and Ethics Committee of Semmelweis University (approval ID: 1895/003/2004).

### Two-electrode voltage clamp and patch clamp measurements

Two-electrode voltage clamp experiments were performed three or four days after the microinjection of cRNA, as previously described [9],. Low [K^+^] solution contained (in mM): NaCl 95.4, KCl 2, CaCl_2_ 1.8, HEPES 5 (pH 7.5 adjusted with NaOH). High [K^+^] solution contained 80 mM K^+^ (78 mM Na^+^ of the low [K^+^] solution was replaced with K^+^). TRESK background K^+^ current was measured at the ends of 250 or 300 ms voltage steps to −100 mV applied in every 4 s.

For current measurements in HEK293 cells, the whole-cell patch clamp technique was applied. The Ca^2+^-free, low [K^+^] extracellular solution had the following composition (in mM): NaCl 140, KCl 2, MgCl_2_ 2.5, glucose 11, EGTA 0.05, HEPES 10, pH 7.4 (adjusted with NaOH). High [K^+^] extracellular solution contained 30 mM K^+^ (28 mM Na^+^ of the low [K^+^] solution was replaced with K^+^). During the stimulation with ionomycin a further version of these extracellular solutions was applied, which contained 2 mM CaCl_2_, 0.5 mM MgCl_2_ and no EGTA. Pipettes were pulled from borosilicate glass by a P-87 puller (Sutter Instrument Co., Novato, CA) and fire polished. Pipette resistance ranged between 4 and 9 MΩ when filled with the solution containing (in mM): KCl 140, MgCl_2_ 3, EGTA 0.05, Na_2_ATP 1, Na_2_GTP 0.1, HEPES 10, pH 7.3 (adjusted with NaOH). In some experiments, ATP and GTP were omitted from the pipette solution. The pipette was connected to the headstage of a patch-clamp amplifier Axopatch-1D (Axon Instruments, Inc., Foster City, CA). Data were filtered at 1 kHz and digitally sampled at 2 kHz by a Digidata 1200 interface board (Axon Instruments). Data acquisition and analysis was performed with pCLAMP 10 software (Molecular Devices, Sunnyvale, CA). The voltage protocol consisted of a 200 ms voltage step to −100 mV followed by a 600 ms ramp to +60 mV applied in every 2 s. TRESK background K^+^ current was analyzed at the end of the voltage steps to −100 mV.

### *In vitro* radioactive phosphorylation

Solution B contained 50 mM Tris-HCl (pH 7.5), 5 mM MgCl_2_, 0.5 mM PMSF and 0.5 mM benzamidine. GST-TRESKloop, GST-TRESKloop-TAPtag and GST-tau proteins immobilized on 10 µl glutathione-agarose were phosphorylated with Trx-His_6_-MARK2-T208E in 50 µl volume of solution B supplemented with 20 µM Na_2_ATP, 100 kBq ^32^P-γ-ATP, 2 mM EGTA and 0.5 mM DTT. The constitutively active, T208E mutant form of MARK2 was used, because this version of the kinase did not require the phosphorylation of T208 in the activation loop. The immobilized substrates were phosphorylated at 30°C for 1 hour with continuous shaking at 200 rpm. The proteins were run on 12% SDS-PAGE gels, the gels were stained with Coomassie Brilliant Blue, and their radioactivity was detected with phosphorimager (GS-525, Bio-Rad, Hercules, CA, USA).

The different TRESKloop-His_8_ constructs immobilized on Ni-NTA resin were phosphorylated with GST-MARK2-T208E in a similar manner. In this case, solution B was supplemented with 1.4 mM β-mercaptoethanol in addition to 20 µM Na_2_ATP and 50 or 100 kBq ^32^P-γ-ATP. The substrate proteins were run on 15% SDS-PAGE gels because of the small size of TRESKloop-His_8_. This highly hydrophobic small protein fragment was weakly stained with Coomassie Blue, and run anomalously at around 19 kD instead of the calculated 13.5 kD molecular weight.

### Statistics and calculations

Data are expressed as means±S.E. Statistical significance was estimated by Student's t test for independent samples, with the exception of a one-way analysis of variance (ANOVA) and *Tukey HSD* post hoc test for multiple comparisons (as indicated in the appropriate figure legend). The Statistica 8.0 program package (StatSoft, Tulsa, OK) was used for the analysis. The difference was considered to be significant at p<0.05.

## Results

### TRESK is activated by the elevation of cytoplasmic calcium concentration via calcineurin in HEK293 cells

**Figure 1:**
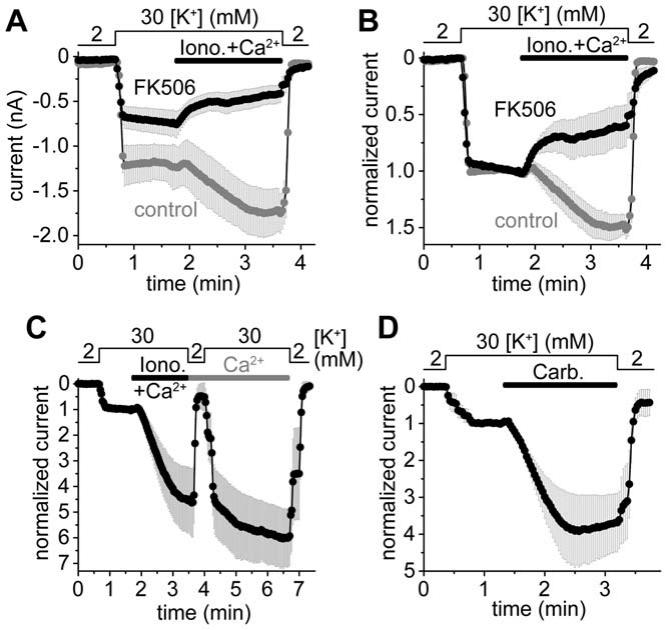
Calcineurin activates mouse TRESK several-fold in HEK293 cells. **A.** Calcium-dependent activation of TRESK current was measured at −100 mV in HEK293 cells after FK506 pretreatment (1 µM, 15–40 min, *FK506, black curve*) or without the application of the calcineurin inhibitor (*control, gray curve*). Whole-cell patch clamp recording was performed with calcium-free pipette and bath solutions containing 50 µM EGTA. Pipette solution contained GTP (0.1 mM) and ATP (1 mM). Extracellular [K^+^] was increased from 2 to 30 mM (as indicated above the graph) and the cells were subsequently challenged with ionomycin (1 µM) plus Ca^2+^ (2 mM, *Iono.+Ca^2+^*, *horizontal black bar*). **B.** Normalized responses to ionomycin plus calcium were calculated from the same recordings as represented in panel A. FK506 prevented the calcium-dependent activation of TRESK. **C.** TRESK currents during the stimulation with ionomycin plus calcium (*Iono.+Ca^2+^*, *horizontal black bar*) were measured with calcium-free pipette solution containing 50 µM EGTA but neither ATP nor GTP. Calcium (2 mM) was continuously present after the withdrawal of ionomycin (*Ca^2+^*, *horizontal gray bar*). Extracellular [K^+^] was repeatedly changed between 2 and 30 mM as indicated above the graph. The currents were normalized to the basal value measured before the stimulation. **D.** TRESK current was stimulated with carbachol (50 µM) via endogenous muscarinic receptors. The pipette and bath solutions were Ca^2+^-free, containing 50 µM EGTA and no ATP/GTP. Normalized curves were plotted; for average current data of panel C and D see Figure S3.

TRESK current in HEK293 cells was measured under whole-cell patch clamp conditions with unusual calcium-free pipette and bath solutions containing EGTA at low (50 µM) concentration. Combined extracellular application of calcium (2 mM) and the calcium-ionophore ionomycin (1 µM) activated TRESK current by 50±12% (n = 6, *gray control* curves, Figure 1.A and B, for further comments on this stimulation see Figure S2). This moderate degree of activation corresponded to previous patch clamp data in the literature [10], [11], and allowed the examination of the mechanism of TRESK activation.

When the cells were pretreated with the calcineurin inhibitor FK506 (1 µM) for 15–40 min, the application of calcium and ionomycin resulted in 38±18% inhibition of the K^+^ current (n = 6, *black FK506* curves, Figure 1.A and B, p<0.01 compared to the control group). This inhibition was likely evoked by the direct pharmacological effect of ionomycin on TRESK, as it has already been described in *Xenopus* oocytes [15]. Pretreatment with the selective calcineurin inhibitor FK506 prevented the calcium-dependent TRESK activation, suggesting that endogenous calcineurin also regulated TRESK in HEK293 cells.

In the above experiment, basal TRESK current appeared to be larger in the control than in the FK506 group (although the difference was not statistically significant, p = 0.09, Figure 1.A). We assumed that despite of the calcium-free solutions TRESK in the control group was still preactivated by our experimental manipulations before recording. Therefore ATP was omitted from the pipette solution, to eliminate possible activation of endogenous purinergic G_q_ protein-coupled receptors [18] by the leakage of ATP from the pipette tip before the formation of gigaseal and the consequent calcium release from intracellular stores. When this pipette solution was applied and an additional period after the stimulation was inserted in order to wash out ionomycin and clearly evaluate the calcium-dependent effect, TRESK current was activated 6.0±1.1-fold (n = 5, Figure 1.C). This degree of activation is close to that characteristic for TRESK in the *Xenopus* oocyte expression system [9].

When TRESK-expressing HEK293 cells were stimulated by the muscarinic agonist carbachol (50 µM) via endogenous receptors, TRESK current was activated 3.9±0.9-fold (n = 5, Figure 1.D). Thus the calcium release from the intracellular stores was sufficient to regulate TRESK when calcium-free pipette and bath solutions were applied. Elevated calcium levels in the physiological range, evoked by endogenous receptors and signaling mechanisms, substantially activated the channel in HEK293 cells.

### Coexpression of MARK2 with TRESK accelerates the return of the K^+^ current to the resting state after the calcium-dependent activation

The sequence of the S274/276/279 cluster does not match the consensus motifs of known serine/threonine kinases. On the basis of different hypotheses, inhibitor sensitivity and consensus sequence similarity (*not shown*) we have cloned and functionally tested 40 wild type (*wt*) or constitutively active (*ca*) kinase constructs (corresponding to 23 different serine/threonine kinase types, see Figure S4). The cRNA of these kinases was coinjected with that of TRESK into *Xenopus* oocytes. The searched-for regulatory kinase was expected to (re)phosphorylate TRESK channel after its calcineurin-mediated dephosphorylation, and accordingly accelerate the return of the K^+^ current to the resting state after the ionomycin-stimulation. However, these kinases failed to accelerate recovery (*data not shown*).

**Figure 2:**
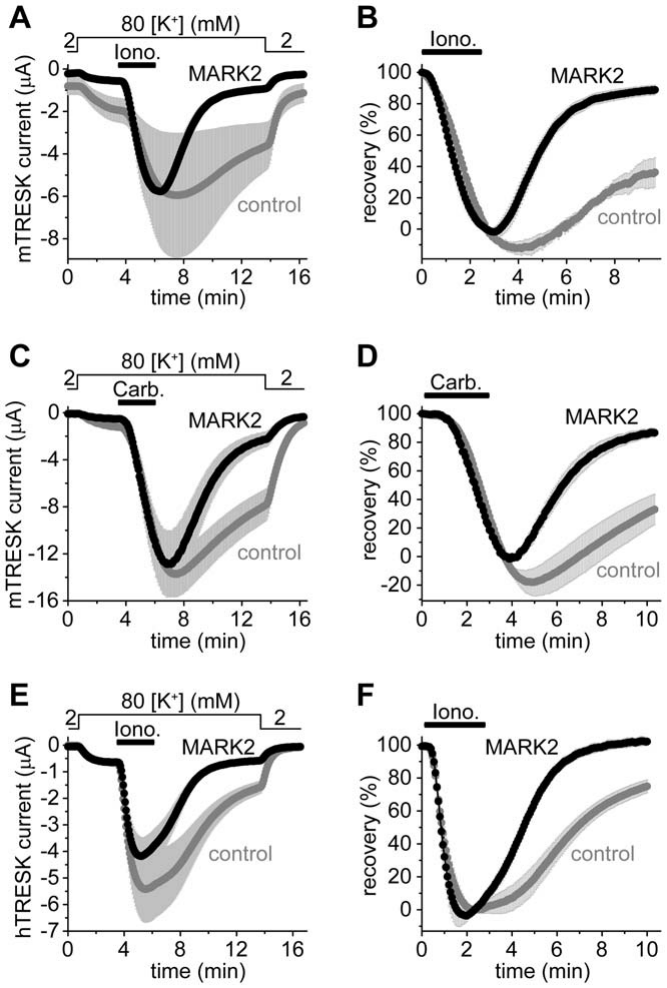
The coexpression of MARK2 with TRESK accelerates the return of the background K^+^ current to the resting state after the calcium-dependent activation. **A.** Background K^+^ currents of *Xenopus* oocytes coexpressing mouse wild type TRESK with MARK2 kinase (*MARK2, black curve*) or expressing only the channel (*control, gray curve*) were stimulated with ionomycin (*Iono.*, 0.5 µM, *horizontal black bar*). Extracellular [K^+^] was changed from 2 to 80 mM and back as indicated above the graph. Note that the resting K^+^ current (the difference between the currents in 2 and 80 mM [K^+^] at the beginning of the measurement) was smaller in the cells coexpressing MARK2 with TRESK than in the control oocytes, whereas the average peak currents after stimulation in the two groups were identical in this experiment. **B.** The recovery of the currents of each oocyte (the same cells as in panel A) was calculated as a percent. The K^+^ current of the oocytes coexpressing MARK2 with TRESK almost completely returned to the resting value in contrast to that of the control cells expressing only the channel. **C.** A similar experiment as in panel A was performed with oocytes coexpressing mouse TRESK and M_1_ muscarinic receptor with MARK2 (*MARK2*, triple coexpression, *black curve*) or without the kinase (*control, gray curve*). The cells were stimulated with carbachol (1 µM, as indicated by the *horizontal black bar*). **D.** Recovery data were calculated from the recordings represented in panel C. MARK2 accelerated the return of K^+^ current to the resting value after receptor stimulation. **E.** The same experiment as in panel A was performed with human TRESK. (For further comments on these results see Figure S5.) **F.** Recovery data were calculated from the currents of panel E. The recovery of human TRESK current to the resting state was accelerated by MARK2 after the calcium-dependent activation.

Recently, we have reached the conclusion that the kinase phosphorylating the S274/276/279 cluster is inhibited by the adaptor protein 14-3-3 [14]. Therefore we have attempted to identify TRESK-inhibitory kinase on the basis of its sensitivity to 14-3-3. We examined microtubule-associated-protein/microtubule affinity-regulating kinase 2 (MARK2), since it was reported to be inhibited by 14-3-3 [19], [20]. In the cells coexpressing MARK2 with mouse TRESK, the K^+^ current almost completely recovered (89±3%, n = 9) after the ionomycin-stimulation at the end of the measurement (Figure 2. A and B). This was in sharp contrast to the control oocytes expressing only the channel (36±9% recovery, n = 6, p<10^−4^). The amplitude of the resting TRESK current, measured before the application of ionomycin, was significantly smaller in the MARK2 (0.34±0.04 µA) than in the control group (1.05±0.17 µA, p<0.01, Figure 2.A). Thus coexpression of MARK2 inhibited TRESK under resting conditions, and dramatically accelerated the (re)phosphorylation of the channel after the calcineurin-dependent activation. Very low amounts of MARK2 cRNA (0.16 ng/oocyte) were sufficient to significantly accelerate TRESK recovery, suggesting that the effect did not rely on massive overexpression of the kinase.

The stimulation of G_q_ protein-coupled receptors activate a complex signaling network and the elevation of cytoplasmic [Ca^2+^] is only a part of the process. Therefore we examined whether MARK2 was able to accelerate TRESK current recovery after the more physiological, but at the same time more complex, receptor stimulation (Figure 2.C and D). In the cells coexpressing TRESK, M_1_ muscarinic receptor and MARK2 (triple coexpression), recovery of the K^+^ current after the withdrawal of carbachol (1 µM) was more rapid (87±3% in the end, n = 10, *black curve*, Figure 2.D) than in the control oocytes coexpressing only the receptor and the channel (33±11%, n = 13, *gray* curve, p<0.001). Thus MARK2 was also effective in the case of receptor stimulation. It is interesting to note that the apparent activation of K^+^ current in the MARK2 group was 30.0±4.9-fold (*black* curve, Figure 2.C). This indicates that TRESK has a remarkable at least 30-fold dynamic range of receptor-mediated regulation.

The major regulatory region is not identical in the mouse (RLSCSILSNLD) and human channel (RLSYSIISNLD). The discrepancy, especially the cysteine to tyrosine substitution, raised the question whether human TRESK was also inhibited by MARK2. Therefore the experiment plotted in Figure 2.A and B was also performed with the human channel (Figure 2.E and F). The recovery was 75±4% (n = 7) in the control and 102±2% (n = 7) in the MARK2 group at the end of the measurement (p<0.001, Figure 2. F). MARK2 accelerated the return of the K^+^ current to the resting state, irrespectively of the different regulatory sequences in the two TRESK orthologs.

### MARK2 inhibits S264E mutant mouse TRESK

**Figure 3:**
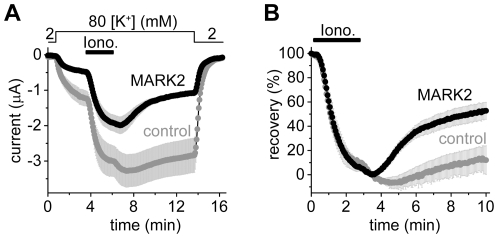
The coexpression of MARK2 accelerates the recovery of the K^+^ current of S264E mutant mouse TRESK after the stimulation with ionomycin. **A.** Average currents of two groups of oocytes coexpressing S264E mutant TRESK with MARK2 kinase (*MARK2, black curve*), or expressing only the S264E mutant channel (*control, gray curve*) were plotted. The cells were stimulated with ionomycin (*Iono.*, 0.5 µM, as indicated by the *horizontal black bar*) in 80 mM extracellular [K^+^] (as shown above the graph). **B.** Recovery was calculated from the same recordings as in panel A. Note the accelerated recovery in the cells coexpressing MARK2 with the S264E mutant channel.

We have previously reported that calcium-dependent TRESK regulation relies on two regions, S264 and the S274/276/279 cluster [14]. In order to elucidate whether S264 was necessary for TRESK regulation by MARK2, we examined the effect of the kinase on the S264E mutant channel (Figure 3). Evidently, MARK2 could not regulate TRESK through the phosphorylation of S264 in this mutant, and the direct recruitment of 14-3-3 to the channel was also abrogated by the mutation.

In accordance with the elimination of one of the regulatory pathways, the activation of the S264E mutant channel in response to ionomycin was smaller (3.1±0.4-fold in the control group) than the about 6-fold activation characteristic for *wt* TRESK under identical conditions [9]. The coexpression of MARK2 accelerated the recovery of the current of S264E mutant TRESK after the ionomycin-stimulation (53±7% recovery at the end of the measurement in the cells coexpressing S264E mutant TRESK with MARK2 (n = 8) vs. 12±12% in the control oocytes expressing only the channel (n = 8), p<0.02, Figure 3.B). Although the current amplitudes in the control group were larger than in the MARK2 group (Figure 3.A), this was not so in a similar experiment performed with MARK2-T208E (where the constitutively active kinase also significantly accelerated the recovery, see Figure S6). Thus MARK2 accelerated the recovery of S264E mutant TRESK irrespectively of the current amplitudes. Serine 264 and the direct interaction of 14-3-3 with TRESK were not indispensable for the effect of MARK2, suggesting that this kinase acted via the S274/276/279 cluster.

### MARK1, MARK2 and MARK3 inhibit TRESK, but not all AMPK-related kinases regulate the channel

**Figure 4:**
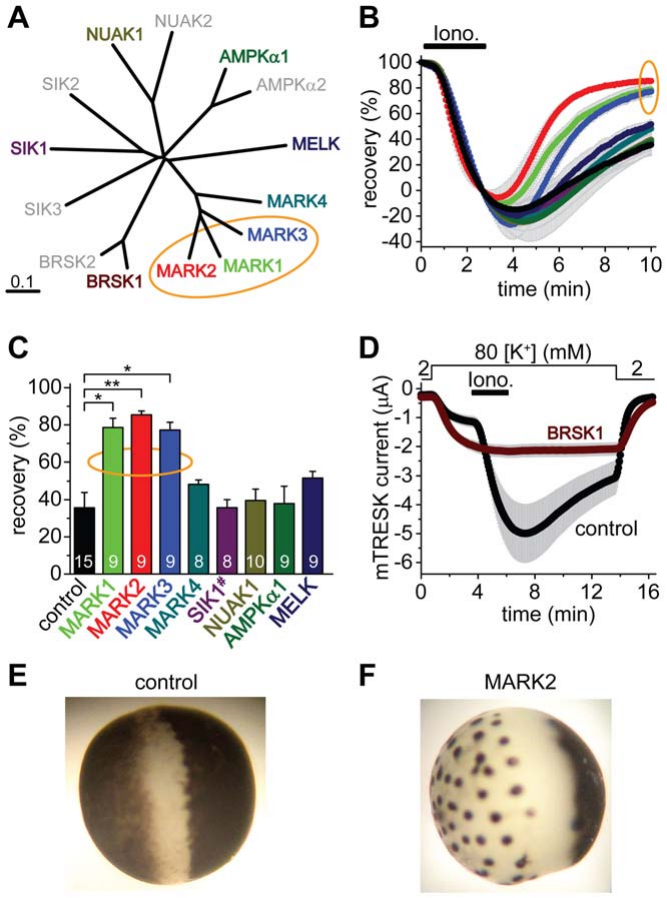
MARK1, 2 and 3 inhibit TRESK, BRSK1 is a possible regulator, whereas the other tested AMPK-related kinases do not influence the recovery of the K^+^ current. **A.** Multiple alignment and phylogenetic tree of mouse full-length AMPK-related kinases was created with Clustal W2 and TreeView. The enzymes, which have been cloned and functionally tested on TRESK, are shown in colors different from gray. The MARK kinases, which efficiently inhibit TRESK, are indicated with an orange ellipse. **B.** Time-dependent recovery of background K^+^ currents after the ionomycin stimulation (*Iono.*, 0.5 µM, as indicated by the *horizontal black bar*) is shown for the groups of oocytes coexpressing the different AMPK-related kinases with mouse TRESK. Color code is the same as in panel A. Rapid recovery of K^+^ current in the MARK1, 2 and 3 groups is indicated with an orange ellipse. **C.** Average recoveries at the end of the measurement are shown for the different groups. The number in the columns indicates the sample size. SIK1 construct (*SIK1^#^*) contained amino acids 1–343, which included the kinase domain. The recovery in the MARK1, 2 and 3 groups was significantly different from that of the control cells (one-way ANOVA, followed by *Tukey HSD* test, *p<0.01, **p<0.001). **D.** Oocytes coexpressing BRSK1 and mouse TRESK (*BRSK1, ferruginous curve, n = 16*) or expressing only the channel (*control, black curve, n = 15*) were stimulated with ionomycin as in the case of the other AMPK-related kinases in panels A, B and C. Note that ionomycin did not activate TRESK current in the cells, which coexpressed BRSK1 with the channel. **E.** Representative photograph of a control oocyte expressing TRESK channels. Appearance is not different from a non-injected cell (*not shown*). **F.** Representative photograph of an oocyte coexpressing TRESK with MARK2 kinase. Note the reduced pigmentation on the animal pole, and the peculiar dark dots on the vegetative hemisphere following a more or less hexagonal arrangement.

We have cloned all four MARK kinases, and one representative member from each family of AMPK-related kinases: AMPKα1, BRSK1 (also called synapses of amphids defective, SAD1, SAD-B), NUAK1, MELK and a SIK1 construct containing amino acids 1–343, including the kinase domain (Figure 4. A). These kinases were coexpressed with mouse *wt* TRESK, and the recovery of the K^+^ current to the resting state after the stimulation with ionomycin was examined.

The three closely related members of the MARK family accelerated the recovery of K^+^ current (Figure 4.B), indicating that MARK1, MARK2 and MARK3 inhibited TRESK channel. MARK4, AMPKα1, NUAK1, MELK and SIK1(1–343) did not significantly influence the recovery (Figure 4.C). The effect of BRSK1 on K^+^ current recovery could not be determined in this experiment, because BRSK1 completely blocked the activation of TRESK (Figure 4.D). When the amount of coinjected BRSK1 cRNA was decreased and limited activation of the channel was allowed, the recovery from this activation was or was not accelerated depending on the amount of BRSK1 cRNA (see Figure S7). Thus it cannot be excluded that BRSK1 also inhibits TRESK. Nevertheless, the complete block of activation (Figure 4.D) was not caused by overwhelming TRESK inhibition; BRSK1 rather interfered with the regulation at multiple points.

Coexpression of MARK or BRSK1 kinases with TRESK induced striking morphological changes of *Xenopus* oocytes, whereas the coexpression of the other AMPK-related kinases did not affect the appearance of the cells. We did not find the description of this morphology in the literature, thus it is possible that we report it for the first time. MARK1, MARK2 and MARK3 reduced the surface area of brown pigmentation at the animal pole, and also resulted in peculiar dark dots on the vegetative hemisphere (compare Figure 4.E and F for MARK2; for further details see Figure S8). The cell biology behind this complex morphology has not been further examined, however, the question was raised whether TRESK inhibition was the consequence of the long-term structural rearrangements induced by MARK kinases or was independent of them.

### Microinjection of constitutively active MARK2 protein acutely inhibited TRESK, and the enzyme activity of the coexpressed kinase was required for TRESK regulation

**Figure 5:**
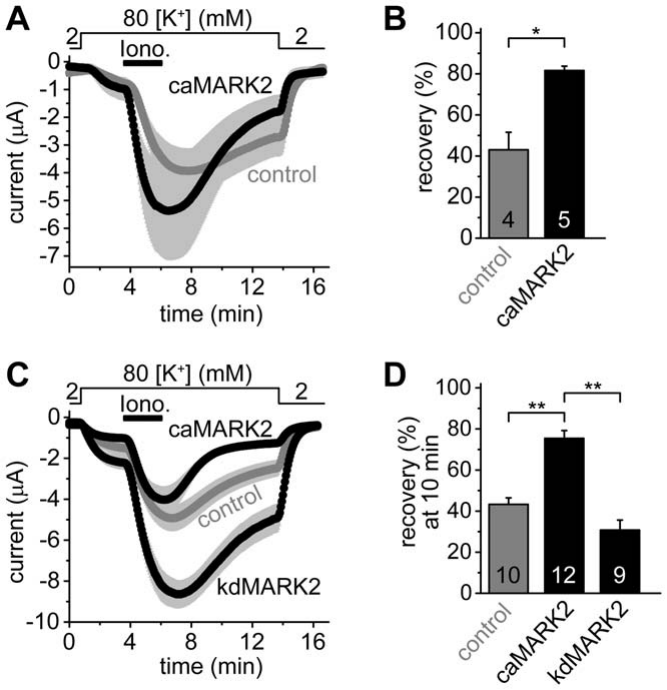
Microinjection of constitutively active MARK2 protein into *Xenopus* oocytes accelerates the recovery of TRESK current to the resting state after ionomycin-stimulation. The coexpression of a 14-3-3-insensitive, constitutively active form of MARK2, but not the kinase-dead version of the enzyme, inhibits TRESK. **A.** *Xenopus* oocytes coexpressing human TRESK with human 14-3-3η were microinjected with the constitutively active, partially 14-3-3-insensitive GST-MARK2-T208E,T539A kinase (*caMARK2, black curve*), or with the heat-inactivated form of the same protein (*control, gray curve*). The cells were stimulated with ionomycin as in Figure 2.A. The microinjection of the proteins was performed 144–169 min before the application of ionomycin. **B.** Average K^+^ current recoveries are shown at the end of the measurement in the groups introduced in panel A. Recovery in the group of oocytes microinjected with the active kinase (*caMARK2*) was significantly accelerated, compared the *control* oocytes (*p<0.002). **C.** Average currents of three groups of oocytes were compared. In the first group, mouse TRESK was coexpressed with 14-3-3-insensitive constitutively active MARK2 (*caMARK2*, MARK2-T208E,S400A,T539A construct, *black curve*,). In the second group, the channel was coexpressed with kinase-dead MARK2 (*kdMARK2*, MARK2-T208A,S212A,S400A,T539A construct, *black curve*). In the third group, only TRESK was expressed (*control, gray curve*). The experimental protocol was the same as in Figure 2.A. **D.** Average recoveries at 10 minutes in the three groups shown on panel C were plotted as indicated below the columns. The K^+^ current recovered more rapidly in the *caMARK2* group than in the *control* or *kdMARK2* cells (**p<10^−5^ for both comparisons with Student's t-test, which is significant at the p<0.05/3 limit according to Bonferroni correction.) The numbers in the bars in panel B and D indicate the number of measured oocytes.

GST-MARK2-T208E,T539A, a constitutively active, partially 14-3-3-insensitive MARK2 protein was produced in *E. coli*. This protein was microinjected 144–169 min before the application of ionomycin into oocytes coexpressing human TRESK with 14-3-3η. (The latter was used to suppress the endogenous kinase phosphorylating TRESK). In the oocytes microinjected with constitutively active MARK2 protein, the recovery of TRESK current to the resting state after the ionomycin-stimulation was accelerated (compared to the control cells microinjected with heat-inactivated MARK2 protein, Figure 5.A and B). Characteristic changes of cellular morphology did not develop during this short period of MARK2-treatment (*not shown*), indicating that long-term structural rearrangements were not required for TRESK inhibition.

As another approach to confirm that the kinase activity of MARK2 was required for TRESK inhibition and MARK2 did not only compete with an endogenous oocyte kinase for 14-3-3, we tested two further modified versions of the enzyme. In these constructs the phosphorylation-dependent binding sites of 14-3-3 were disrupted by S400A and T539A mutations [19]–[21]. In order to obtain constitutively active (*ca*) or kinase-dead (*kd*) constructs, T208E or T208A/S212A mutations were additionally introduced [22], [23]. The coexpression of MARK2-T208E,S400A,T539A (*caMARK2*, Figure 5.C and D) accelerated the recovery of TRESK current after the ionomycin-stimulation (p<10^−5^, compared to either the control oocytes expressing only TRESK (*gray control* curve and column, Figure 5.C and D) or the *kd*MARK2 group). MARK2-T208A,S212A,S400A,T539A (*kdMARK2*) did not accelerate TRESK recovery (Figure 5.C and D). Since *ca*MARK2 inhibited TRESK but *kd*MARK2 did not evoke this effect, the kinase activity of MARK2 was indispensable for TRESK regulation.

### MARK2 directly phosphorylates the S274/276/279 cluster but does not affect S264

**Figure 6:**
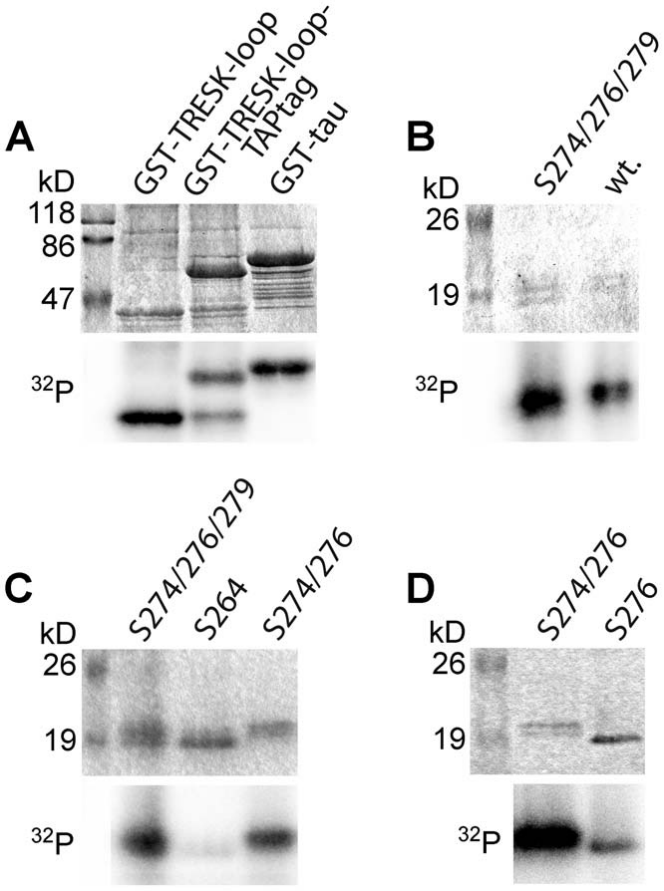
MARK2 directly phosphorylates the S274/276/279 cluster of mouse TRESK in vitro. **A.** GST-TRESKloop, GST-TRESKloop-TAPtag or GST-tau (positive control) fusion proteins were phosphorylated with constitutively active Trx-His_6_-MARK2-T208E in the presence of [γ−^32^P]ATP. The upper panel shows the SDS-PAGE gel stained with Coomassie Blue, whereas the autoradiogram of the same gel is on the lower panel. The two GST-fusion constructs containing amino acids 185–292 of mouse TRESK were labeled with ^32^P to a similar intensity as the GST-tau control (see the *lower^32^P* panel). In the GST-TRESKloop-TAPtag sample, an incompletely translated (or degradation) product (slightly larger than GST-TRESKloop in the other lane, see the *upper* panel) was also phosphorylated. **B.** Wild type TRESKloop-His_8_ (*wt.*) or the mutant version of this protein containing only S274, S276 and S279 (*S274/276/279 lane*) were phosphorylated with GST-MARK2-T208E. Note that the substrate containing only the three serines of the S274/276/279 cluster was also strongly labeled with ^32^P. **C.** The mutant TRESKloop-His_8_ substrates, containing only the serines indicated above the lanes, were phosphorylated with GST-MARK2-T208E. Both substrates retaining S274 and S276 (*S274/276/279* and *S274/276 lanes*) were labeled with ^32^P, in contrast to the protein containing only serine 264 (*S264 lane*). **D.** The mutant TRESKloop-His_8_ substrates, containing both serine 274 and 276 (*S274/276 lane*) or only serine 276 (*S276 lane*) were phosphorylated with GST-MARK2-T208E. Note that the S276 substrate was labeled with ^32^P, although to a lesser extent than the protein retaining both S274 and S276.

We examined whether the intracellular loop region of TRESK was phosphorylated by MARK2 *in vitro*. To compare the phosphorylation of TRESK with that of a well-known substrate of the kinase, we have cloned a version of the microtubule-associated protein tau and produced it as a GST-fusion construct (GST-tau). This version of tau contained three repeat domains including the KXGS motifs. (KXGS is the generally-used substrate sequence in MARK2 kinase assays.) The loop region of TRESK was produced in two forms as GST-TRESKloop or GST-TRESKloop-TAPtag fusion proteins, both containing amino acids 185–292 of mouse TRESK. The substrate proteins were immobilized on glutathione-agarose beads, and Trx-His_6_-MARK2-T208E, the thioredoxin-hexahistidine-tagged constitutively active MARK2 kinase was added in the presence of [γ-^32^P]ATP. The GST-TRESKloop substrates were similarly labeled with ^32^P as the positive control GST-tau under identical assay conditions (Figure 6.A), indicating that the intracellular loop of TRESK was efficiently phosphorylated by MARK2.

In the tag-reversed experiment, TRESKloop-His_8_ substrates, immobilized on Ni-NTA agarose, were phosphorylated with GST-MARK2-T208E kinase. Wild type TRESKloop-His_8_ contained 10 serines and 1 threonine, including S264 and the S274/276/279 cluster. (In further experiments we focused on these four regulatory serines, because we have previously demonstrated that mutation of the other residues did not interfere with TRESK regulation in *Xenopus* oocytes [9].) In addition to wild type TRESKloop-His_8_, a mutant version of this substrate was also tested, which contained only the three serines of the S274/276/279 cluster, whereas the other 7 serines and threonine were replaced with alanine. GST-MARK2-T208E phosphorylated both wild type TRESKloop-His_8_ (*wt*, lane 2, Figure 6.B) and the substrate protein containing only the S274/276/279 cluster (*S274/276/279*, lane 1, Figure 6.B). In conclusion, the S274/276/279 cluster is a direct target of MARK2-mediated phosphorylation.

Another TRESKloop-His_8_ construct was used to evaluate whether MARK2 phosphorylated S264. In this construct, all the serines and threonine residues, except S264 were mutated. This substrate was not phosphorylated by GST-MARK2-T208E (*S264*, lane 2, Figure 6.C). Thus the specificity of MARK2 for the two regulatory sites in TRESK is complementary to that of protein kinase A. Protein kinase A phosphorylates S264 but not the S274/276/279 cluster [15], whereas MARK2 phosphorylates the S274/276/279 cluster but not S264.

In the KXGS substrate sequence of tau, the phosphorylated serine is in the +3 position from the positively charged lysine residue. The RLSCSILS sequence of TRESK does not follow the same rule. While S274 and S276 (positions +2 and +4 from the positively charged arginine) are adjacent to position +3, S279 (+7) is far away from it. Accordingly, the TRESKloop-His_8_ construct containing only S274 and S276 (*S274/276*, lane 3, Figure 6.C) was similarly phosphorylated as the substrate containing all three serines of the S274/276/279 cluster (lane 1, Figure 6.C). This suggests that S279 is not a major target of MARK2. (The TRESKloop-His_8_ construct containing only S279 was not phosphorylated by MARK2 (*not shown*). However, this may also be the consequence of impaired interaction between the enzyme and the S274A/S276A double-mutant substrate.).

It is not feasible to distinguish the phosphorylation of S274 from that of S276 by mutational analysis, since the mutation of one of these residues likely interferes with the phosphorylation of the other. Nevertheless, we pursued the phosphorylation of S276 further, because the mutations of this residue evoked the most substantial effect on TRESK in *Xenopus* oocytes; the S276A and S276C mutants were constitutively active channels [9]. The TRESKloop-His_8_ construct containing only S276 was also phosphorylated by MARK2, although to a lesser extent than the substrate retaining both S274 and S276 (Figure 6.D). This weak phosphorylation of the S274A mutant protein still suggests that S276 is a target of MARK2 in the native substrate sequence. Irrespective of the distribution of phosphorylation between the two residues, S274 and S276 together are efficiently phosphorylated by MARK2. These in vitro results with the unequivocal functional data in *Xenopus* oocytes suggest that the phosphorylation of S274 and S276 is directly responsible for the regulatory effects of MARK kinases on TRESK in the living cell.

## Discussion

Enzymatic interaction between MARK and TRESK is novel in the sense that no other ion channel is known to be phosphorylated by this kinase, and the connection between the functions of MARK [24] and those of TRESK [25] has not been suspected. The identification of this kinase/substrate pair raises the question whether TRESK is related to the regulation of cell polarity [26], axonal/dendritic differentiation [27]–[30] and microtubule dynamics [22] in the specific neuron populations where the channel is expressed [8], [10], [12], [31]–[33]. The intensively studied and presently emerging signaling pathways, which control MARK (for review see [34], [35]), may also regulate the background K^+^ conductance of the plasma membrane and neuronal excitability.

The interaction between MARK and TRESK is highly specific. More than 20 other serine/threonine kinase types failed to inhibit TRESK current via the S274/276/279 cluster when they were functionally tested in *Xenopus* oocytes. Among them were several enzymes of wide substrate specificity (such as protein kinase A, protein kinase C, casein kinase 2, etc.), also including kinases often responsible for the regulation of other ion channels. The number of possible kinase candidates for TRESK regulation was significantly reduced [36], [37], when the negative effect of 14-3-3 on the TRESK-regulatory kinase was recognized in the *Xenopus* system [14]. We have found MARK on the basis of this observation. Because MARK kinases are widely-expressed regulators of cellular polarity, they are likely present and phosphorylate TRESK in the cells, which express the channel. The mRNA of a MARK kinase (MARK3) is specifically transported to and translated in axons of adult DRG neurons [38].

In *Xenopus* oocytes, TRESK is activated several-fold by calcineurin. However, in mammalian cells, only limited TRESK activation (30–100%) was observed, and the mechanism of TRESK regulation has not been previously examined [10], [11]. In the present study, we found that the selective calcineurin inhibitor FK506 prevented TRESK activation in HEK293 cells. (Another inhibitor, cyclosporin A (0.5 µM) also blocked TRESK activation when endogenous muscarinic receptors were stimulated; *results not shown*.) Thus the pivotal role of endogenous calcineurin in TRESK activation has been verified in mammalian cells. General regulation of TRESK by calcineurin is in good accordance with the previous result that TRESK and calcineurin are associated via direct protein-protein interaction [17].

We have shown for the first time that several-fold TRESK activation can be evoked in mammalian cells, if appropriate Ca^2+^- and ATP-free solutions are applied under whole-cell patch clamp conditions. This indicates that robust TRESK activation is not restricted to the *Xenopus* oocyte expression system. Instead, special conditions are required for its detection in mammalian cells. Particular care must be taken to avoid unwanted and at the same time also allow stimulated elevation of [Ca^2+^] during whole-cell patch clamp measurements. The application of Ca^2+^- and ATP-free solutions is not a diversion from physiology, since these experimental conditions are required to preserve the basal phosphorylation characteristic for TRESK channels in cultured HEK293 cells. The analysis of kinase effects in mammalian cells and the investigation of TRESK regulation in isolated neurons were beyond the scope of the present study. The conditions of whole-cell recording optimized for TRESK activation (no ATP, low Ca^2+^-buffering) do not adequately support the recovery phase, although the preceding activation is necessary for the measurement of current recovery. Further methodological improvements are also required to distinguish TRESK current from the other endogenous background K^+^ currents in native cells.

High stability of long recordings and unperturbed cytoplasmic composition during two-electrode voltage clamp of *Xenopus* oocytes were suitable for the investigation of kinase effects on TRESK regulation. Using this test system, we examined, which relatives of MARK2 can regulate the channel. Distribution of TRESK-regulatory MARK-like kinases within the AMPK-related kinase family did not respect the boundaries of nomenclature and sequence similarity. While MARK1, 2 and 3 undoubtedly regulated TRESK, the closely related MARK4 did not inhibit the channel even if high cRNA amounts were microinjected. In turn, it could not be excluded that BRSK1, which was more apart from the effective MARK kinases on the phylogenetic tree than MARK4, also regulated TRESK. BRSK and MARK kinases are functionally related; they share several substrates among the microtubule-associated proteins (e.g. tau, MAP2 and MAP4) and have overlapping roles in the determination of neuronal polarity [34], [35]. In contrast, the function of MARK4 deviates from those of the other three MARK kinases; MARK4 directly binds to tubulin, and it is accordingly localized to the cellular microtubule network and to centrosomes [39].

Several well-established roles of MARK kinases are intimately related to the plasma membrane. In mammalian epithelial cells, MARK2 is localized to the lateral membrane, but it is excluded from the apical region [40]. Under the apical membrane, MARK2 is phosphorylated by atypical protein kinase C (aPKC), binds 14-3-3 and detaches to the cytoplasm [19], [20]. This mechanism stabilizes the steady-state subcellular localization of MARK2 and contributes to the maintenance of cellular polarity in both epithelial cells and neurons [26]. It has recently been reported that KA1 (kinase-associated 1) domain of MARK binds phosphatidylserine, and can directly attach the kinase to the lipid bilayer [41]. Because MARK kinases can dynamically associate to the plasma membrane, their in vivo localization is compatible with the regulation of ion channels.

Serine 264 and the S274/276/279 cluster are the primary determinants of calcineurin-dependent TRESK regulation; the mutation of other intracellular serine and threonine residues does not interfere with the mechanism [9], [14]. MARK2 accelerated the return of K^+^ current to the resting state after the calcium-dependent activation, even if serine 264, the 14-3-3 binding site of mouse TRESK, was mutated. This indicates that MARK2 does not act via the phosphorylation of S264 and the recruitment of 14-3-3 to the channel in the living cell, but targets the other regulatory region, the S274/276/279 cluster. On the basis of our results, phosphorylation of other intracellular residues of TRESK by MARK kinases also can not be ruled out, however, these residues may have less impact on the channel activity. Phosphorylation of (unknown) TRESK-regulatory proteins by MARK is also possible, especially because modulation of multiple target proteins is the general theme in known kinase-mediated regulations of ion channels [42]. Evidently, further studies are required for the complete elucidation of TRESK regulation by MARK kinases. Nevertheless, the MARK2-induced acceleration of current recovery of S264E mutant TRESK is in good accordance with the in vitro specificity of the kinase for the S274/276/279 cluster.

While the constitutively active MARK mutants accelerated TRESK current recovery, the kinase-dead version failed to do so. It is essential to point out that phosphorylation is required, and the effect does not rely only on protein-protein interactions. We have previously shown that the recovery kinetics of TRESK is profoundly affected by the level of 14-3-3 adaptor protein in the cytoplasm [15]. However, MARK kinases did not act via the reduction of the amount of functionally available 14-3-3, and the consequent stimulation of endogenous TRESK-regulatory kinase activity. Overexpression of 14-3-3 did not eliminate the effect of MARK on TRESK (Figure 5.A and Figure S6). Moreover, the S400A/T539A double-mutation, which was reported to ablate 14-3-3 binding to MARK [21], did not interfere with the effect of the enzyme on the channel (Figure 5. C and D). Thus, MARK does not work as a 14-3-3-scavanger, and its kinase activity is absolutely required for TRESK regulation.

After six years of testing the coexpression of more than 20 kinases with TRESK, several inhibitors and known kinase-activating experimental manipulations without any effect on TRESK recovery in the *Xenopus* system, we have finally found a kinase, which phosphorylates the S274/276/279 cluster and also regulates the channel. It is evidently beyond our resources to test all the known serine/threonine kinases in order to examine whether there are other TRESK-regulatory kinase types. On the basis of our experimental data and irrespective of these other putative kinases, we conclude that heterologously-expressed MARK inhibits TRESK background potassium channel in *Xenopus* oocytes. This finding has major physiological importance if TRESK is similarly regulated by MARK kinases in some neuronal cell types. Furthermore, it is tempting to speculate that TRESK may be related to the regulation of neuronal polarity and/or microtubule cytoskeleton. It is generally accepted that MARK kinases control these systems [24], [34], [35], and we have now shown that they also phosphorylate TRESK. Thus, it is plausible to assume that the regulation of TRESK is connected to the well-established functions of MARK. In addition, preliminary data from other independent experiments, currently in progress in our laboratory, are also consistent with these conclusions. We hope that we will be able to provide further insight into the coordination of cellular polarity, microtubule cytoskeleton and TRESK in the following years.

In summary, we have demonstrated that MARK directly phosphorylates TRESK in vitro and specifically inhibits the channel in *Xenopus* oocytes. These results connect two presently emerging fields and suggest that the control of cellular polarity and microtubule dynamics is coupled to the regulation of background K^+^ current in the cells which express TRESK.

## Supporting Information

Figure S1 **Oligonucleotide sequences and annealing temperatures for touchdown RT-PCR and in vitro site-directed mutagenesis.**
(PDF)

Figure S2 **TRESK activation in response to elevation of extracellular calcium concentration and subsequent application of ionomycin in HEK293 cells.**
(PDF)

Figure S3 **Average currents corresponding to the normalized curves in** **Figure 1.C and D** **.**
(PDF)

Figure S4 **Ineffective wild type and constitutively active kinase constructs tested on TRESK regulation in** ***Xenopus*** **oocytes.**
(PDF)

Figure S5 **Rate of recovery is not determined by the peak current amplitudes in** **Figure 2.E** **.**
(PDF)

Figure S6 **The effect of different constitutively active MARK2 constructs on the current recovery of TRESK-S264E in the presence or absence of overexpressed 14-3-3.**
(PDF)

Figure S7 **Complex modulation of TRESK regulation by BRSK1 coexpression.**
(PDF)

Figure S8 **Changes of pigmentation in** ***Xenopus*** **oocytes coexpressing TRESK with different AMPK-related kinases.**
(PDF)
