# Supplementary material for: TRESK Background K+ Channel Is Inhibited by PAR-1/MARK Microtubule Affinity-Regulating Kinases in Xenopus Oocytes
Source: PLoS One. 2011 Dec 1;6(12):e28119. doi: 10.1371/journal.pone.0028119 (PMC3228728; doi:10.1371/journal.pone.0028119)
Supplement: Figure S1 — Oligonucleotide sequences and annealing temperatures for touchdown RT-PCR and in vitro site-directed mutagenesis. (PDF) [file pone.0028119.s001.pdf]

S1. supplementary information

Oligonucleotide sequences for RT-PCR and *in vitro* site-directed mutagenesis.

Start codons are in italic. Restriction enzyme sites for cloning or those introduced by silent mutations for identifying mutant clones are underlined. Mutations are marked with lowercase letters.

Primer	Sequence (5' → 3')	Restriction enzyme
AMPKα1-s	CCTGGTCCGGAGCCGCCACC*ATG*CGCAGACTCAGTTCCTGG	Kpn2I
AMPKα1-a	GCGCTCGAGCCAGTCCCTGTGCAAACTGCATTCC	XhoI
BRSK1-s	CAGGAATTCGCCGCCACC*ATG*TCGTCCGGGTCCAAGGAAGG	EcoRI
BRSK1-a	CGCGAGATCTGGGGTCAGGGTAGAGGGGTTC	BglII
MARK1-s	GCGCAATTGCCGCCACC*ATG*TCGGCGCGGACGCCATTG	MunI
MARK1-a	GCGCTCGAGTTACAGCTTAAGCTCATTTGCTATTTTGGATG	XhoI
MARK2-s	GCGCAATTGATACCGGCGCC*ATG*TCCAGC	MunI
MARK2-a	ATACCGTCGACTTAAAGCTTCAGCTCATTGGCTATTTTGGAG	SalI
MARK3-s	CCTGGTCCGGAGCCGCCACC*ATG*TCCACTAGGACCCCTTTGC	Kpn2I
MARK3-a	CGCGAGATCTTTACAGCTTTAGCTCATTGGCAATTTTGG	BglII
MARK4-s1*	CCCAGAGAAG*ATG*TCTTCGAGGAC	-
MARK4-s2*	CAGGAATTCGCCGCCACC*ATG*TCTTCGAGGACGGCGCTGGCC	EcoRI
MARK4-a	ATACCGTCGACTCAGAGTTCGAGGTCGTTGGAGATGC	SalI
MELK-s	GCGCAATTGCCGCCACC*ATG*AAAGATTATGACGAACTCCTC	MunI
MELK-a	GCGCTCGAGTCACATCTTGCAGCCAGACAAG	EcoRI
NUAK1-s	CAGGAATTCGCCGCCACC*ATG*GAAGGGGCGGCAGTGTCCGC	EcoRI
NUAK1-a	GCGCTCGAGCTAGTTGAGCTTGCTGCAGATCTCCAGCG	XhoI
SIK1-s	CAGGAATTCGCCGCCACC*ATG*GTGATCATGTCGGAGTTCAG	EcoRI
SIK1-343a	ATACCGTCGACCTACTCGAGTAGGAGGTAGTAAATGG	SalI
Tau-s	CAGGAATTCGCCGCCACC*ATG*GCTGACCCTCGCCAGGAG	EcoRI
Tau-a	GCGCTCGAGCTGCCCTGGGAGCCTGATCAC	XhoI
MARK2-T208E-s**	CTTTGGGAACAAGCTTtGATgaaTTCTGTGGCAGTCC	HindIII
MARK2-T208A/S212A-s**	GGAACAAGCTGGATgCaTTCTGTGGagcTCCTCCTTATGCTG	Mph1103I, SacI
MARK2-S400A-s**	CACAAGGTTCAGCGgtcCGTCgccGCCAACCCCAAGCAAC	Cfr13I
MARK2-T539A-s**	GTGTCCAGTCGAAGCgccTTCCATGCTGG	Bsp143II

(*MARK4-s1 was used in the first 19 cycles, but MARK4-s2 in the following 17 cycles, after a 8.5-fold dilution of the first reaction, for better PCR specificity. **Antisense primers for mutations were the reverse complements of the shown sense oligonucleotides.)

Annealing temperatures and DMSO concentrations for touchdown RT-PCR.

The PCR protocol: 2 min initial denaturation at 98 °C; 36 cycles of (40 sec denaturation at 98 °C, 60 sec annealing, 3 or 4 min extension at 72 °C); 7 min extension at 72 °C. RT product was 4 % of reaction volume.

	AMPKα1	BRSK1	MARK1	MARK2	MARK3	MARK4	MELK	NUAK1	SIK1 (1-343)	Tau
1st 3 cycles	65 °C	67 °C	62 °C	65 °C	62 °C	67 °C	62 °C	67 °C	62 °C	67 °C
2nd 3 cycles	61 °C	64 °C	59 °C	62 °C	59 °C	64 °C	59 °C	64 °C	58 °C	64 °C
3rd 3 cycles	57 °C	61 °C	56 °C	59 °C	56 °C	61 °C	56 °C	61 °C	54 °C	61 °C
Last 27 cycles	55 °C	58 °C	53 °C	57 °C	53 °C	58 °C[#]	53 °C	58 °C	52 °C	58 °C
DMSO	-	10 %	2 %	2 %	2 %	10 %	2 %	2 %	-	2 %

([#]Annealing temperature was increased to 62 °C in the last 17 cycles of the second reaction with MARK4-s2.)
