# Supplementary material for: TRESK Background K+ Channel Is Inhibited by PAR-1/MARK Microtubule Affinity-Regulating Kinases in Xenopus Oocytes
Source: PLoS One. 2011 Dec 1;6(12):e28119. doi: 10.1371/journal.pone.0028119 (PMC3228728; doi:10.1371/journal.pone.0028119)
Supplement: Figure S2 — TRESK activation in response to elevation of extracellular calcium concentration and subsequent application of ionomycin in HEK293 cells. (PDF) [file pone.0028119.s002.pdf]

S2. supplementary information

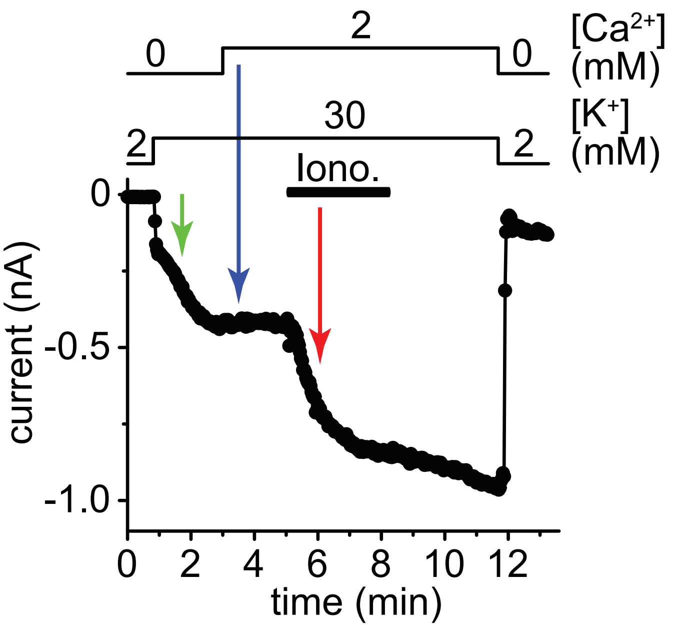

If extracellular (EC) [Ca^{2+}] was elevated from 0 to 2 mM before the application of the calcium-ionophore ionomycin (Iono., 1 μM), then TRESK current was activated by the elevation of [Ca^{2+}] in some HEK293 cells, but only by ionomycin in the others. To circumvent this biological variability, we administered calcium and ionomycin together for reliable TRESK activation in further experiments. It is important to note, however, that in some cells the elevation of [Ca^{2+}] alone did not influence TRESK current at all (*blue arrow*, see the representative recording above). This indicates that the elevation of EC [Ca^{2+}] did not directly influence channel activity through a biophysical mechanism. For example, the "screening" effect of Ca^{2+} ions on the negatively charged phospholipid bilayer, which so profoundly affects the activation of voltage-gated channels, and the binding of the ion to the channel protein, could not be responsible for TRESK activation. This was also verified by the insensitivity of activated TRESK current to the reduction of EC [Ca^{2+}] from 2 to 0 mM (*not shown*). Instead of a direct biophysical effect of EC calcium on the channel, the ion entered the cytoplasm and activated TRESK via calcineurin.

It is also apparent in the figure shown above that TRESK current was small at the beginning of the measurement (less than 0.25 nA, when EC [K^+] was increased from 2 to 30 mM), but the K^+ current slowly increased in the calcium-free EC solution (*green arrow*). This activation was not negligible compared to that evoked by ionomycin (*red arrow*). We elevated EC [Ca^{2+}] only when the K^+ current stabilized (*blue arrow*). The initial activation of TRESK current in the calcium-free EC solution also supported the hypothesis that TRESK was preactivated by the experimental manipulations before the start of recording. The final phase of this preactivation could be detected at the beginning of the measurement. Because no EC Ca^{2+} was present at this time, calcium-dependent activation of the channel was possible through the release of the ion from intracellular stores. This brought ATP into suspicion as an agonist of G_q protein-coupled receptors, leaking from the patch pipette tip before seal formation.
