# Supplementary material for: TRESK Background K+ Channel Is Inhibited by PAR-1/MARK Microtubule Affinity-Regulating Kinases in Xenopus Oocytes
Source: PLoS One. 2011 Dec 1;6(12):e28119. doi: 10.1371/journal.pone.0028119 (PMC3228728; doi:10.1371/journal.pone.0028119)
Supplement: Figure S3 — Average currents corresponding to the normalized curves in Figure 1.C and D . (PDF) [file pone.0028119.s003.pdf]

S3. supplementary information

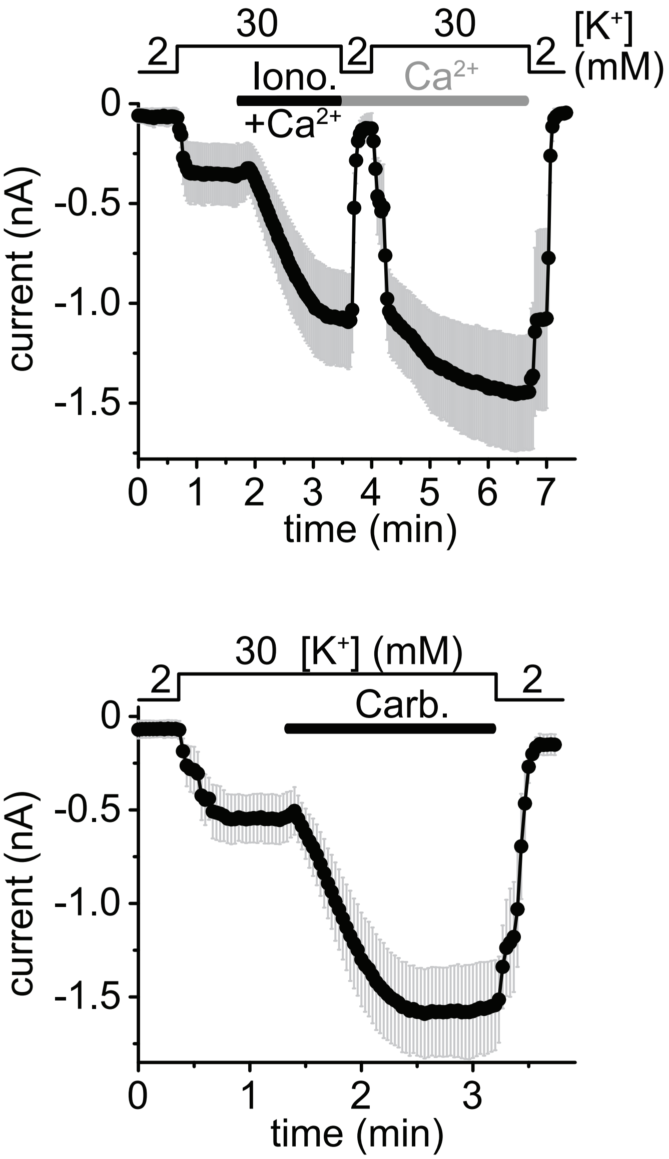

The above two panels show average currents corresponding to the normalized curves plotted in Fig. 1. C and D, respectively. Current amplitudes after the stimulation (with ionomycin or carbachol) were sufficiently large, verifying that heterologously expressed TRESK currents were measured. Endogenous currents of non-expressing HEK293 cells during ionomycin-stimulation were much smaller (*not shown*).
