# Supplementary material for: TRESK Background K+ Channel Is Inhibited by PAR-1/MARK Microtubule Affinity-Regulating Kinases in Xenopus Oocytes
Source: PLoS One. 2011 Dec 1;6(12):e28119. doi: 10.1371/journal.pone.0028119 (PMC3228728; doi:10.1371/journal.pone.0028119)
Supplement: Figure S4 — Ineffective wild type and constitutively active kinase constructs tested on TRESK regulation in Xenopus oocytes. (PDF) [file pone.0028119.s004.pdf]

S4. supplementary information

Kinase construct	Type	Sequencing
AMPKα1	wt	full
AMPKα1-T183D	ca	1-860
AMPKα1(1-324AA)	ca	-
AMPKα1(1-324AA)-T183D	ca	full
B-Raf1	wt	1-974 & 1530-end
B-Raf1-Δ1-426,R427M	ca	full
B-Raf1-Δ1-464,R465M	ca	full
CamKIIβ	wt	-
CamKIIβ-T287D	ca	-
CASK	wt	full
CASK-Δ1-373,V374M	ca	1-820
CASK-S24D+V26L	ca	-
CDK1	wt	-
CDK1-T14A,Y182E	ca	1-883
CHK1(1-352AA)*	ca	full
CHK1(1-416AA)*	ca	full
CHK2	wt	full
CK1α	wt	1-752
CK2α1	wt	full
CK2α2	wt	-
CK2β**	wt	-
C-Raf1	wt	1-906 & 987-end
C-Raf1-Δ1-319,P320M	ca	full
ERK2	wt	full
GRK2	wt	1-978 & 1182-end
GSK3β	wt	1-812
GSK3β-S9A	ca	1-958
MAPKAP-K2-kinase domain-T208E	ca	-
MEK1-S218,222E	ca	full
PKA***	wt	-
PKB-Δ1-128	ca	-
PKB-Δ1-128 with N-terminal Src-myristoylation-sequence	ca	-
PKC (conventional)****	wt	-
PKCζ	wt	full
PKCζ-A119E	ca	-
PLK1	wt	full
PLK1-T210D	ca	1-1078
SIK(1-343AA)	tr	full
SIK(1-343AA)-T182E	ca	-
TGFβR I	wt	full
TGFβR II	wt	full
TGFβR I-T204D	ca	full

The above wild type (*wt*) or constitutively active (*ca*) kinase constructs were functionally tested on TRESK regulation in *Xenopus* oocytes. The coding sequences were amplified by RT-PCR, cloned to pXEN vector, and modified by QuikChange mutagenesis or other standard molecular biological methods to obtain *ca* constructs. Most constructs were verified by automatic sequencing (as indicated in the table). In some cases, only the 5' end of the insert corresponding to the kinase domain was sequenced or two sequencing reactions were performed from the two ends. The quality of all *in vitro* synthesized cRNAs was verified on denaturing agarose gels before their microinjection.

The effect of the two *ca* CHK1 constructs* on TRESK recovery could not be unequivocally determined, since these kinases inhibited TRESK activation. Coexpression of CK2α1 or CK2α2 catalytic subunits with CK2β** regulatory subunit was also tested. (GST-CK2α1 kinase, produced in *E. coli,* phosphorylated milk proteins, but not the S276/274/279 cluster of mouse TRESK *in vitro*.) PKA*** protein was purchased from Sigma. PKA phosphorylated S264 but not the S276/274/279 cluster of TRESK (see Czirják *et. al.*, JBC 2008&2010). Conventional PKC**** isoforms have not been cloned, but their activator, the phorbol ester PMA failed to influence TRESK recovery, when PKC activation was unequivocally verified in the oocytes.
