# Supplementary material for: TRESK Background K+ Channel Is Inhibited by PAR-1/MARK Microtubule Affinity-Regulating Kinases in Xenopus Oocytes
Source: PLoS One. 2011 Dec 1;6(12):e28119. doi: 10.1371/journal.pone.0028119 (PMC3228728; doi:10.1371/journal.pone.0028119)
Supplement: Figure S5 — Rate of recovery is not determined by the peak current amplitudes in Figure 2.E . (PDF) [file pone.0028119.s005.pdf]

S5. supplementary information

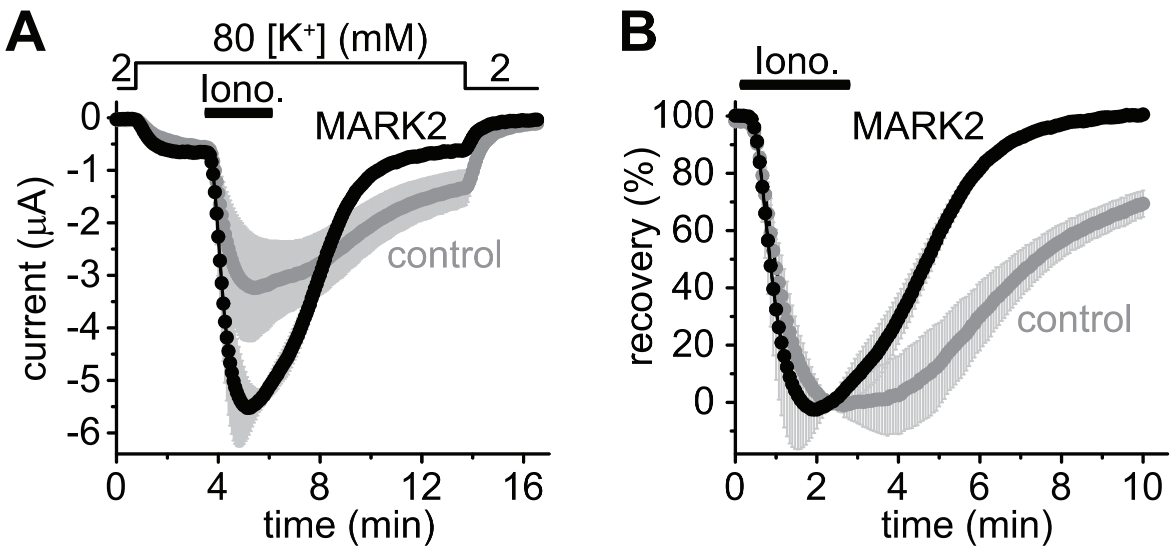

In Fig. 2. E, the average peak current of the oocytes coexpressing human TRESK with MARK2 was smaller than in the control cells expressing only the channel. This may raise the question whether the quick recovery of K^+ current in the MARK2 group was the consequence of small current amplitudes. In order to address this question, the 4 cells with the smallest peak currents were selected from the control group, and they were compared to the 4 cells with the largest peak currents in the MARK2 group (see *panel A and B* above; the calculations were performed in the same manner as in Fig. 2. E and F). In this analysis, the average peak current in the MARK2 group was larger than in the control group (*panel A*), however, the recovery was more rapid in the cells coexpressing the kinase with the channel than in the control oocytes (*panel B*, $p<0.01$ at the end of the measurement). This indicates that MARK2 accelerates the return of human TRESK current to the resting state after the stimulation with ionomycin, irrespectively of the peak current amplitudes.
