# Supplementary material for: TRESK Background K+ Channel Is Inhibited by PAR-1/MARK Microtubule Affinity-Regulating Kinases in Xenopus Oocytes
Source: PLoS One. 2011 Dec 1;6(12):e28119. doi: 10.1371/journal.pone.0028119 (PMC3228728; doi:10.1371/journal.pone.0028119)
Supplement: Figure S6 — The effect of different constitutively active MARK2 constructs on the current recovery of TRESK-S264E in the presence or absence of overexpressed 14-3-3. (PDF) [file pone.0028119.s006.pdf]

S6. supplementary information

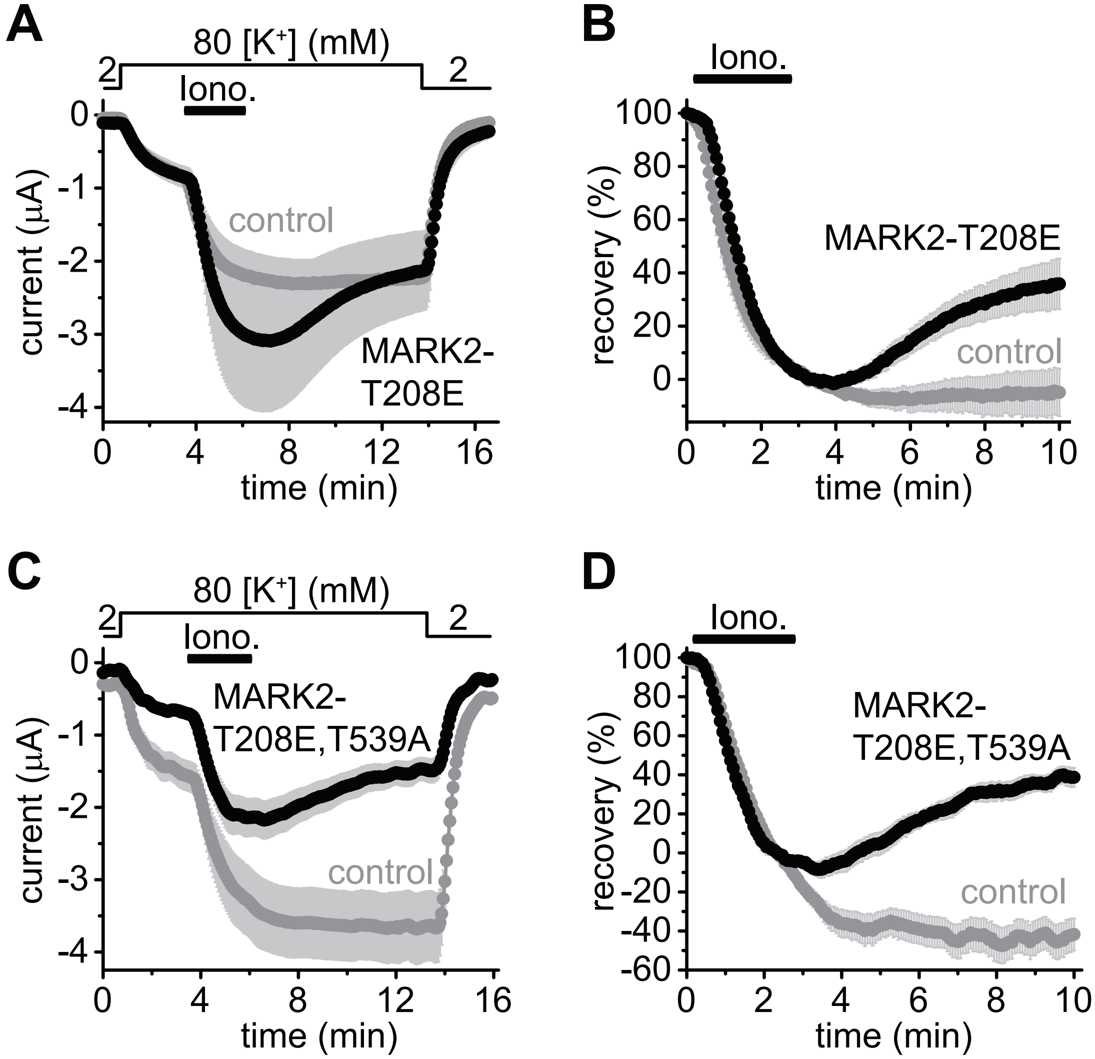

A. Average currents of two groups of oocytes coexpressing (mouse) S264E-mutant TRESK with constitutively active T208E-mutant MARK2 kinase (*MARK2-T208E, black curve*), or expressing only the S264E mutant channel (*control, gray curve*) were plotted. The cells were stimulated with ionomycin (*Iono.*, 0.5 μM, as indicated by the *horizontal black bar*) in 80 mM extracellular [K^+] (as shown above the graph). In contrast to the experiment shown in Fig. 3., the average peak current of the cells coexpressing the kinase with the channel (*black*) was larger than that of the control oocytes (*gray*). **B.** Recovery was calculated from the same recordings as in panel A. Recovery at the end of the measurement was 36±9 % in the cells coexpressing S264E mutant TRESK with MARK2-T208E (n=4) vs. -5±9 % in the control oocytes (n=6, p<0.02). Irrespectively of the amplitude of average peak currents, the recovery of S264E-mutant TRESK was accelerated by the kinase. **C.** A similar experiment as in panel A was performed with the partially 14-3-3-insensitive, constitutively active form of the kinase, MARK2-T208E,T539A. In this experiment, 14-3-3η was also overexpressed in both groups, in order to inhibit the endogenous kinase of the oocyte. **D.** Recovery was calculated from the same recordings as in panel C. Recovery at the end of the measurement was 39±5 % in the cells coexpressing S264E mutant TRESK and 14-3-3η with *MARK2-T208E,T539A* (n=13) vs. -42±8 % in the *control* oocytes coexpressing S264E mutant TRESK with 14-3-3η (n=14, $p<10^{-7}$, the negative value of recovery indicated that the current increased further after the washout of ionomycin in the control group.) Overexpression of 14-3-3 did not eliminate the effect of the (partially 14-3-3-insensitive) coexpressed kinase on TRESK, suggesting that MARK2 did not act via the reduction of the functional availability of endogenous 14-3-3.
