# Supplementary material for: TRESK Background K+ Channel Is Inhibited by PAR-1/MARK Microtubule Affinity-Regulating Kinases in Xenopus Oocytes
Source: PLoS One. 2011 Dec 1;6(12):e28119. doi: 10.1371/journal.pone.0028119 (PMC3228728; doi:10.1371/journal.pone.0028119)

S7. supplementary information

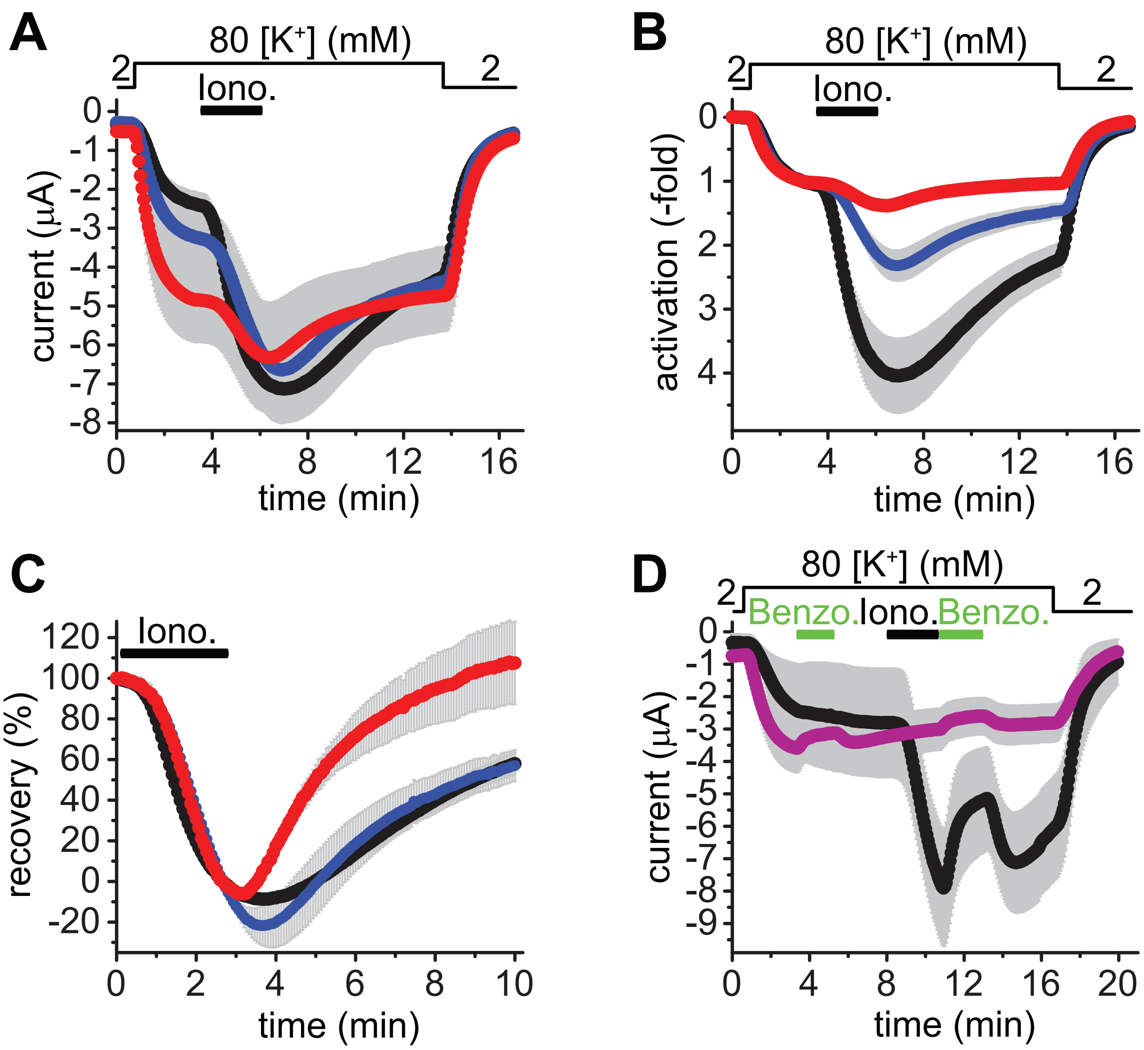

A. Average currents of three groups of oocytes are shown. Different amounts of BRSK1 cRNA (*red curve*: 170 pg/oocyte, n=5; *blue curve*: 56 pg/oocyte, n=7) were coinjected with that of TRESK (330 pg/oocyte), while the control cells expressed only the channel (*black curve*, n=14). The cells were stimulated with ionomycin (*Iono.*, 0.5 µM, as indicated by the *horizontal black bar*) in 80 mM extracellular [K^+] (as shown above the graph). Note that the coexpression of BRSK1 increased the basal TRESK current (in 80 mM EC [K^+], before ionomycin-stimulation, 4.8±1.1 µA vs. 2.3±0.8 µA, $p<0.01$). This is the opposite of the effect that would be expected for a kinase simply inhibiting TRESK. **B.** Apparent activation of TRESK current in the same oocytes as in panel A. (Current curves were normalized to the basal value before the stimulation.) At these microinjected cRNA amounts, BRSK1 did not completely diminish TRESK activation, in contrast to the 0.5 ng/oocyte injection shown in Fig. 4. D. **C.** Recovery was calculated from the same recordings as in panel A. Recovery was accelerated by the coexpression of BRSK1 if 170 pg kinase cRNA was microinjected but not by the injections of 56 pg (58±5 % vs. 108±20 %, $p<0.01$; recovery was 57±8 % in the ‘low kinase’ group). Since the recovery calculated as a percent is the most appropriate parameter for the estimation of kinase activity (e.g. recovery in absolute units or rate of recovery are theoretically inadequate), these results suggest that TRESK is inhibited by BRSK1. Nevertheless, the small amplitude of current activation in response to ionomycin (1.6±0.4 µA, panel A) makes this conclusion somewhat uncertain. (continued on the next page.)

S7. (continued)

D. The reduced apparent activation of TRESK (panel B) may reflect that BRSK1 interferes with the mechanism of activation or that TRESK channels are basally active before the stimulation. In order to discriminate between these two possibilities, the sensitivity of basal TRESK current to benzocaine (Benzo., 1 mM) was examined. Benzocaine distinguishes between activated TRESK and the resting channel in *Xenopus* oocytes (*Czirják et al.*, JBC 2006). The basal current is weakly inhibited by benzocaine, compared to the current after the stimulation with ionomycin (*Iono.*, 0.5 µM, see the *black curve* of control oocytes expressing only TRESK, n=3). The current of oocytes coexpressing TRESK with BRSK1 (0.5 ng/oocyte, *purple curve*, n=5) was not activated in response to ionomycin, however, its sensitivity to benzocaine was intermediate between those of the resting and the activated channels. This suggests that a higher fraction of TRESK channels is active under resting conditions in the presence of BRSK1 than in the control cells (in good accordance with the increased basal TRESK current in the cells coexpressing BRSK1 with the channel, see in panel A). However, this alone does not explain the complete loss of activation. BRSK1 may also interfere with the mechanism of activation. In summary, BRSK1 seems to affect TRESK regulation at least in three ways: it increases the fraction of active channels under resting conditions, it interferes with the mechanism of calcium-dependent activation, and may also inhibit the channel after the calcium-dependent activation.

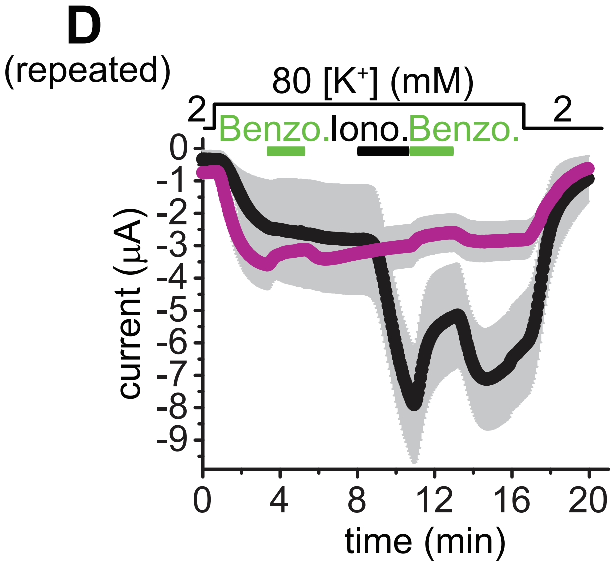

Supplement: Figure S7 — Complex modulation of TRESK regulation by BRSK1 coexpression. (PDF) [file pone.0028119.s007.pdf]
