# Supplementary material for: TRESK Background K+ Channel Is Inhibited by PAR-1/MARK Microtubule Affinity-Regulating Kinases in Xenopus Oocytes
Source: PLoS One. 2011 Dec 1;6(12):e28119. doi: 10.1371/journal.pone.0028119 (PMC3228728; doi:10.1371/journal.pone.0028119)
Supplement: Figure S8 — Changes of pigmentation in Xenopus oocytes coexpressing TRESK with different AMPK-related kinases. (PDF) [file pone.0028119.s008.pdf]

S8. supplementary information

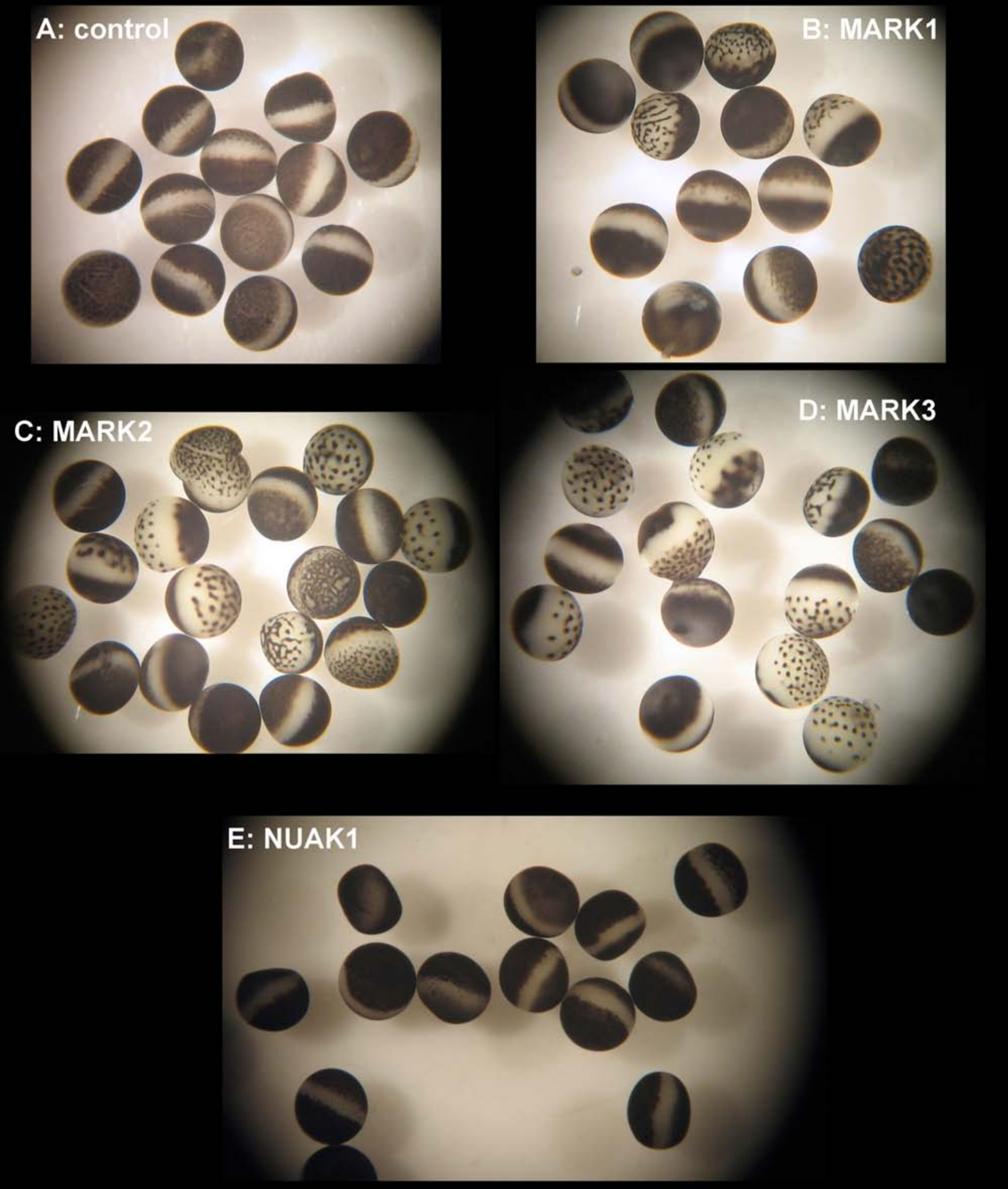

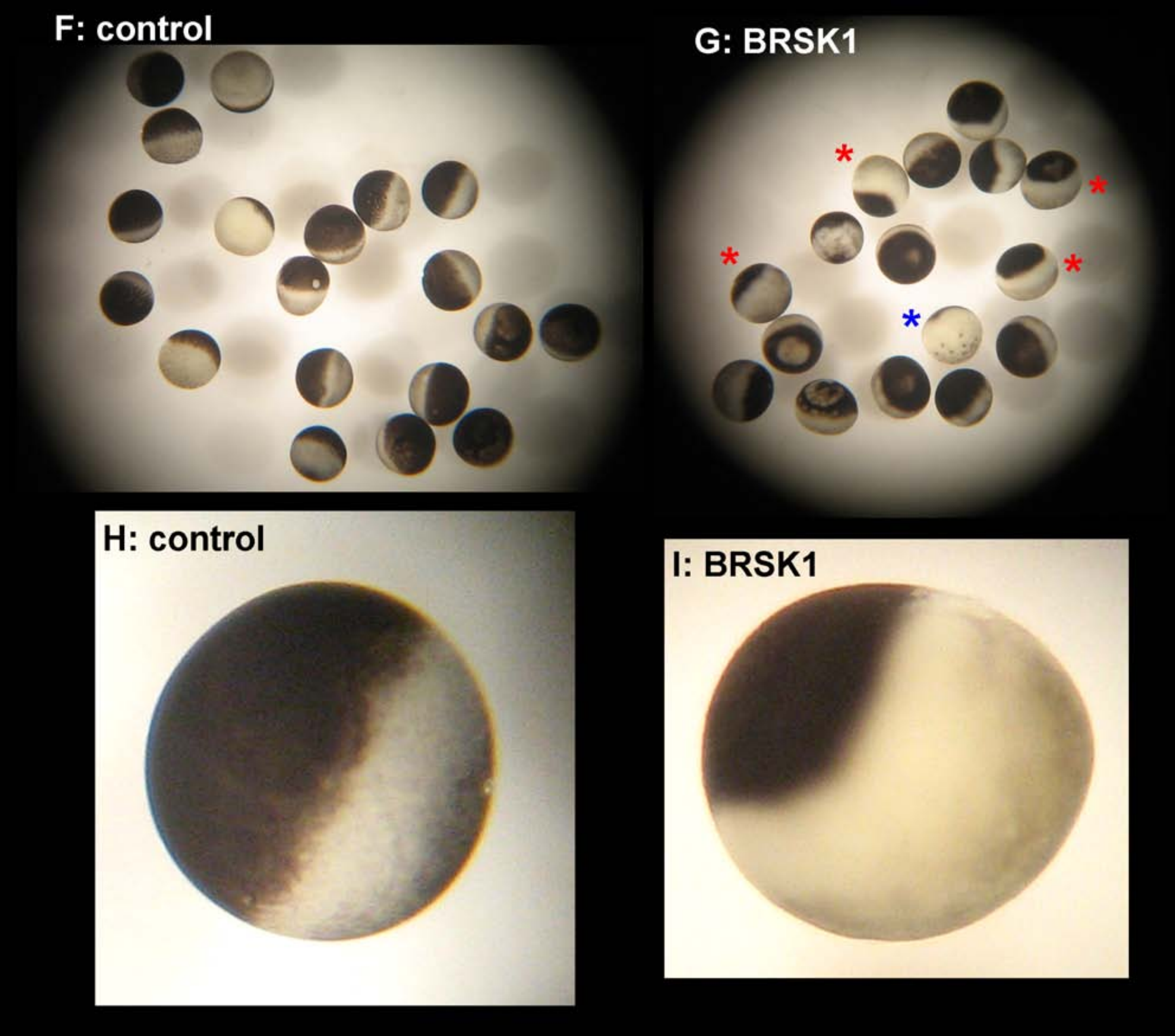
F: control
G: BRSK1
H: control
I: BRSK1

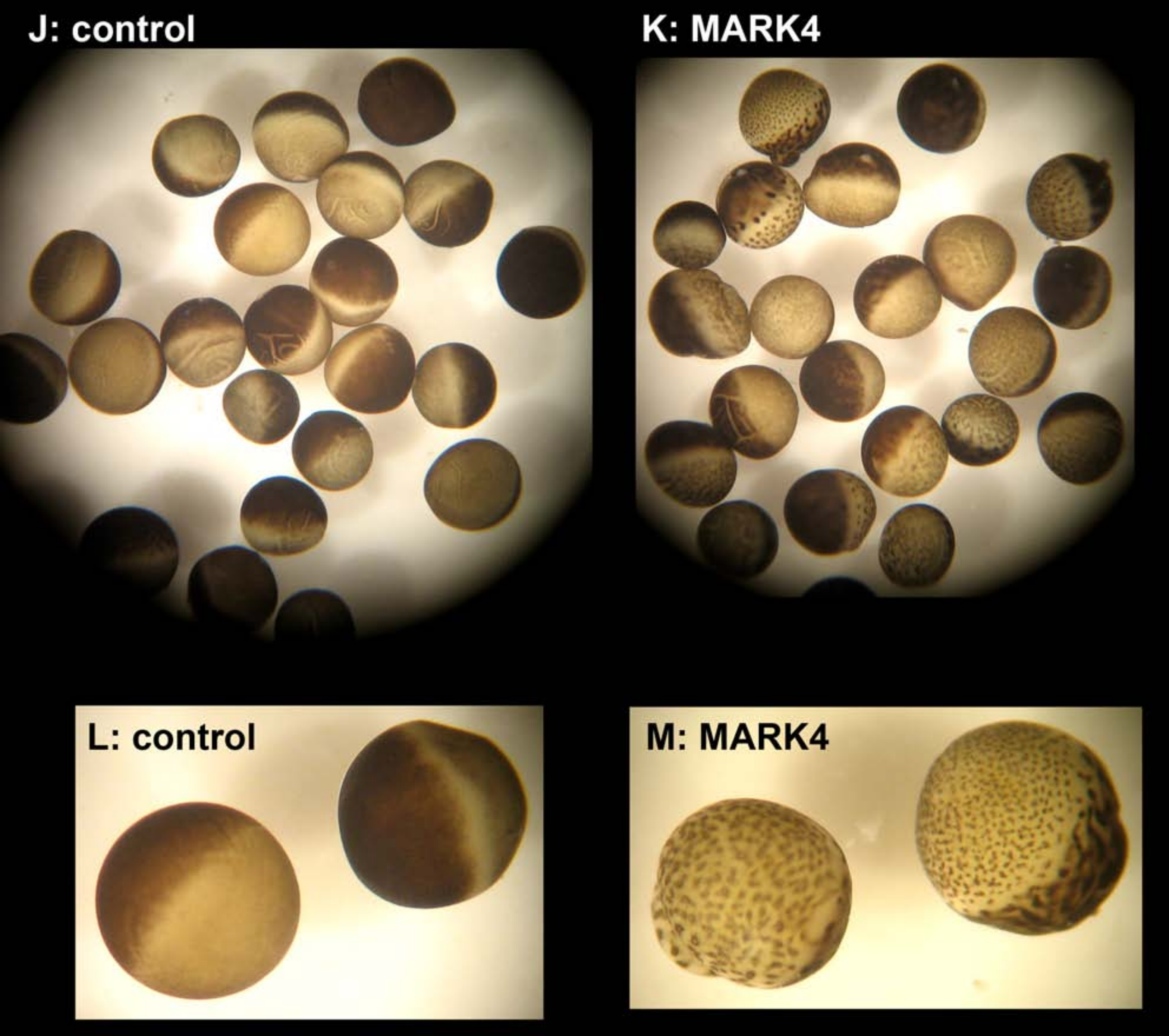
J: control
K: MARK4
L: control
M: MARK4

S8. (continued)

In the above three sets of photographs, visible morphological changes induced by different AMPK-related kinases are illustrated. The general appearance of control oocytes, expressing mouse TRESK (*panel A, F, J*, corresponding to three oocyte preparations, respectively), could not be distinguished from that of the non-injected cells (*not shown*). In the cells coexpressing TRESK with MARK1, 2 or 3 (see *panel B, C* or *D*, respectively), the morphology was strikingly changed. The surface area of brown pigmentation at the animal pole was reduced, and dark dots developed on the vegetative hemisphere. The dots followed a more or less hexagonal arrangement in some oocytes; they were randomly scattered or coalesced to stripes or more elaborate patterns in some other cells. While the reduction of the pigmentation at the animal pole may be the consequence of the shift of balance between the aPKC- and MARK-dominated regions of the plasma membrane (corresponding to the apical and lateral membranes of epithelial cells, respectively, *Böhm et al.* 1997 and *Hurov et al*. 2004, see refs. in the main text), the origin of the dark dots is more enigmatic, and may be related to the microtubule cytoskeleton. Since the main issue of our study was TRESK regulation, we did not engage in the investigation of the cellular mechanisms responsible for the special morphology of MARK-expressing oocytes. Nevertheless, the phenomenon visually verified the expression of the kinases. It is important to note that the typical dotted morphology developed only in a fraction of the cells, but TRESK current recovery was accelerated both in the cells of dotted appearance and in those, which did not show this characteristic alteration.

In the oocytes coexpressing TRESK with AMPKα1, MELK, NUAK1 or SIK1(1-343), no visible change was observed. We show this only for NUAK1 in *panel E*.

The morphology induced by the coexpression of BRSK1 with TRESK was different from that obtained with MARK kinases. The brown pigmentation was similarly reduced as in the case of MARK coexpression (compare *panel F* and *G*, see the cells marked with *red stars* in *panel G*). However, the dark dots did not (*red stars*) or only rarely (*blue star* in *panel G*) developed. The oocytes with a small brown cap of animal pole pigmentation and a large clear yellowish belly of the extended vegetative hemisphere (see *panel I*) were quite different from both the control cells (*panel H*) and those expressing MARK kinases.

Since the coexpression of MARK4 (0.5 ng/oocyte cRNA) with TRESK did not result in a significant acceleration of TRESK current recovery (Fig. 4. C), the coinjection of higher amount of this kinase cRNA (2.5 ng/oocyte) was also tried in another experiment. Although this high amount of MARK4 cRNA also failed to induce the acceleration of TRESK recovery (*not shown*), the effect of the kinase on visible morphology could be documented (compare *panel J* to *K*, and *L* to *M*). The reduction of the brown pigmentation at the animal pole was not so conspicuous, and the dots seemed to be more numerous, fainter, smaller and more diffuse than in the cells coexpressing TRESK with MARK1, 2 or 3 (*panel B, C* or *D*). Irrespective of the cause of this discrepancy (e.g. the deviation of the biological function of MARK4 from the other MARK kinases or the different oocyte preparations), the morphological change verified the expression of the kinase, and indicated that MARK4 was not effective on TRESK.
